# The Impact of Business Coaching on Founder Confidence and Entrepreneurial Orientation in University Incubated Startups

**DOI:** 10.12688/f1000research.175927.1

**Published:** 2026-05-09

**Authors:** Jacobus Wiwin Kuswinardi, Moses Laksono Singgih, Achmad Affandi

**Affiliations:** 1 School of Interdisciplinary Management and Technology, Institut Teknologi Sepuluh Nopember, Surabaya, East Java, Indonesia; 2 Department of Industrial and Systems Engineering, Institut Teknologi Sepuluh Nopember, Surabaya, East Java, Indonesia; 3 Electrical Engineering Department, Institut Teknologi Sepuluh Nopember, Surabaya, East Java, Indonesia

**Keywords:** Business coaching, locus of control, self-efficacy, entrepreneurial orientation, university business incubators, entrepreneurship

## Abstract

**Background:**

Drawing on Social Cognitive Theory, the research addresses a critical gap concerning how environmental support mechanisms in developing-country incubators foster entrepreneurial mindsets and strategic behaviors. This study aims to examine how business coaching within university business incubators shapes the cognitive and behavioral orientations of student founders in Indonesia by analyzing its influence on locus of control, self-efficacy, and entrepreneurial orientation.

**Method:**

A quantitative research design was employed, involving 220 student founders whose startups were incubated in university business incubators across Indonesia. Data were collected through structured questionnaires and analyzed using PLS-SEM to test both direct and mediating effects among variables.

**Results and Conclusion:**

The results show that business coaching significantly enhances founders’ locus of control, self-efficacy, and entrepreneurial orientation. Both locus of control and self-efficacy also exhibit strong positive effects on entrepreneurial orientation, confirming their roles as central cognitive mechanisms underpinning entrepreneurial behavior. Furthermore, mediation analysis reveals that locus of control and self-efficacy significantly mediate the relationship between business coaching and entrepreneurial orientation. These findings underscore the importance of coaching not merely as a technical intervention but as a psychological and developmental process that strengthens founders’ agency, confidence, and strategic entrepreneurial posture within university incubation environments. This study offers novel empirical evidence on the psychological mechanisms through which business coaching influences entrepreneurial orientation in university incubators within a developing-country context—a setting often overlooked in the literature.

## 1. Introduction

In recent decades, University Business Incubators (UBIs) have emerged as key enablers that facilitate and accelerate the development of new ventures, particularly those vulnerable to uncertainty in dynamic business environments ( Wu et al., 2025). UBIs also function as an essential mechanism for strengthening the entrepreneurial university ecosystem by embedding entrepreneurship education and practice into the core functions of higher education institutions ( Etzkowitz, 2003; Longva, 2021). A well-developed entrepreneurial ecosystem within universities enhances their role not only in fostering innovation and entrepreneurial activity ( Wu et al., 2020) but also in contributing to job creation and regional economic development, rather than focusing solely on producing graduates ( Guerrero et al., 2020, 2024). The support provided by UBIs to increase the likelihood of new venture success includes technical, professional, and financial assistance ( Leitão et al., 2022), access to networked environments ( Wu et al., 2020; 2024), and structured business coaching ( Crompton et al., 2012; Kotte et al., 2021). Consequently, business incubators play a critical intermediary role in developing entrepreneurial capabilities and enhancing the resilience of startups founded by young entrepreneurs ( Branca et al., 2025; Wu et al., 2025). Moreover, the capacity and capability of young founders to navigate uncertainty serve as determining factors in shaping their sense of control and emotional stability, both of which are essential for sound decision-making and long-term business sustainability ( Leonelli et al., 2025; Utomo et al., 2025).

Previous studies have highlighted that startup success rates remain exceedingly low—often below 10% ( Chakraborty et al., 2023; Krishna et al., 2016)—mainly because startups must contend with various internal risk factors, including founders’ demographic characteristics, human capital, and the quality of business planning ( Bielialov, 2022). Leonelli et al. (2025) emphasize that startups frequently overlook the behavioral complexities required to sustain business resilience. In addition, startups often neglect essential aspects of developing entrepreneurial orientation and founder mindset, both of which are critical determinants of success within the industry ( Hung Kee & Rahman, 2017; Utomo et al., 2025). Understanding the inherent traits of young entrepreneurs—particularly university students—remains underexplored in the entrepreneurial behavior literature, as noted by Utomo et al. (2025). Moreover, prior studies examining university-based new ventures in both developed and developing countries remain limited ( Mohammadi et al., 2025). These gaps indicate an unresolved research problem in the entrepreneurial behavior literature concerning the extent to which entrepreneurial traits and behaviors influence startup success. Meanwhile, Crompton et al. (2012) have shown that business coaching facilitated by business incubators can enhance entrepreneurs’ self-confidence and self-efficacy. Business coaching focuses on skills development and active learning processes that help cultivate a growth mindset and self-reflection ( Kotte et al., 2021; Marvel et al., 2020; Somia et al., 2024).

Stimulating self-reflection requires the role of business coaching, which acts as an intervention within entrepreneurial practices for startups led by young entrepreneurs ( Behrendt & Greif, 2018). Business coaching emphasizes developmental interventions that leverage collaborative, reflective, and goal-oriented relationships to achieve valuable outcomes ( Behrendt & Greif, 2018; Kotte et al., 2021). Page and Holmström (2023) further highlight critical aspects of university business incubator experiences in delivering business coaching services, particularly in market research, managerial guidance, and business planning, which are essential to startup success. Crompton et al. (2012) explored the role of business coaching in enhancing entrepreneurs’ self-confidence and self-efficacy within incubator programs, demonstrating its potential to improve performance and achieve goals. The interplay among business coaching, self-confidence, and self-efficacy among young entrepreneurs has been evidenced in several studies that show strong interconnections (e.g., Arkorful & Hilton, 2022; Maxheimer & Nicholls-Nixon, 2022; Nair & Blomquist, 2021; Nicholls-Nixon & Maxheimer, 2022). Moreover, Hossain and Valliere (2024) emphasize that entrepreneurial passion and self-efficacy must be actively addressed through business coaching interventions, as these factors can significantly influence startup failure rates.

Another key construct relevant to this study is entrepreneurial orientation, a firm-level strategic posture characterized by innovativeness, proactiveness, and risk-taking ( Lumpkin & Dess, 1996). Entrepreneurial orientation reflects the extent to which startups pursue innovation, anticipate market opportunities, and commit resources to uncertain ventures ( Chen et al., 2023; Daradkeh and Mansoor, 2023; Donbesuur et al., 2020). Numerous studies have established a strong link between entrepreneurial orientation and business performance across different contexts, including SMEs and startups ( Daradkeh and Mansoor, 2023; Donbesuur et al., 2020; Rosado-Cubero et al., 2024). In the specific context of university business incubators, entrepreneurial orientation refers to how founders’ strategic mindset and orientation toward entrepreneurship translate the support received from coaching into organizational behavior and performance outcomes ( Blank, 2023). Founders with a high entrepreneurial orientation are more likely to leverage coaching to drive innovation, seize opportunities, and differentiate their ventures in competitive markets ( Rosado-Cubero et al., 2024). In incubator contexts, this translates into a greater capacity to internalize and apply external knowledge (from coaches and networks) to entrepreneurial decision-making. This provides strong support for the notion that university incubators do more than provide infrastructure — they actively shape nascent entrepreneurs’ strategic orientation.

Integrating these constructs—business coaching, locus of control, self-efficacy, and entrepreneurial orientation—offers a comprehensive framework for understanding how both external support and internal founder attributes shape startup success in university incubators. From a theoretical standpoint, this study is grounded in Bandura’s (2001) social cognitive theory, which emphasizes the interplay between personal factors, behavior, and environmental influences. Business coaching represents the ecological support system provided by UBIs, while locus of control and self-efficacy capture the cognitive and motivational dimensions of the founders. Entrepreneurial orientation, in turn, reflects the behavioral manifestation of these internal and external influences, shaping how the startup operates and competes in the market. Therefore, examining the interrelationships among these variables contributes to a more holistic understanding of entrepreneurial development within academic incubation ecosystems. As an emerging economy with a growing entrepreneurial ecosystem, Indonesia has witnessed significant policy initiatives to promote university-based entrepreneurship. Programs such as the Program Pengembangan Kewirausahaan (PPK) and Startup Innovation Program under the Ministry of Education and Culture encourage universities to develop business incubators and integrate entrepreneurship into academic curricula. Understanding how business coaching influences these aspects can inform more effective incubation strategies tailored to founders’ psychological and developmental needs.

## 2. Literature review and hypotheses development

### 2.1 Social cognitive theory

Literature on the development of student-led startups within University Business Incubators (UBIs) highlights that university-based incubation plays a fundamental role in shaping the behaviors, capabilities, and strategic orientations of young entrepreneurs. UBIs provide technical, managerial, and psychosocial support, including business coaching specifically designed to strengthen reflective skills, decision-making abilities, and preparedness for uncertainty ( Kotte et al., 2021; Page & Holmström, 2023). Coaching is viewed as a developmental intervention that exposes student-founders to real business practices and encourages self-regulated learning and strategic adaptation ( Marvel et al., 2020; Somià et al., 2024). In the context of student startups—where founders often lack significant business experience—coaching serves as a catalyst that enhances cognitive, emotional, and behavioral capacities ( Arkorful & Hilton, 2022; Maxheimer & Nicholls-Nixon, 2022). Theoretically, the relationships among these variables can be explained through Social Cognitive Theory (SCT) ( Bandura, 2001). SCT posits that human behavior is shaped by the triadic reciprocal interaction among personal factors (cognitive and affective processes), environmental influences, and behavioral outcomes. In this study, business coaching serves as an ecological determinant, providing support, feedback, and social learning opportunities. Locus of control and self-efficacy represent personal cognitive mechanisms that shape how founders process information, develop confidence in their abilities, and assess their capacity to act. Entrepreneurial orientation, in turn, is the behavioral outcome arising from the interaction between founders’ psychological conditions and the environmental support provided by UBIs.

Two psychological attributes frequently associated with the effectiveness of coaching in the incubation process are locus of control and self-efficacy. Founders with a strong internal locus of control tend to believe that their actions determine their venture’s success, making them more responsive to the learning processes facilitated by coaches and incubators ( Newman et al., 2019). Meanwhile, self-efficacy has been consistently shown to influence risk-taking, opportunity evaluation, and resilience in the face of failure ( Baron and Hmieleski, 2018; Leonelli et al., 2025). UBIs play an essential role in strengthening self-efficacy by providing mentoring, business simulations, and exposure to supportive entrepreneurial environments ( Nair & Blomquist, 2021; Rosado-Cubero et al., 2024). Both characteristics shape the extent to which founders can effectively utilize incubation resources, including networking opportunities and business model validation. Beyond individual psychological factors, the literature underscores the importance of entrepreneurial orientation (EO) as a determinant of student startup success. EO—reflecting innovativeness, proactiveness, and risk-taking—has been widely recognized as a key antecedent of startup performance ( Chen et al., 2023; Daradkeh & Mansoor, 2023). Within university environments, EO is influenced by the interaction between founder attributes and incubation support ( Rosado-Cubero et al., 2024). UBIs strengthen EO through knowledge transfer, coaching interventions, and access to professional networks ( Blank, 2023). Therefore, EO can be viewed as a behavioral manifestation resulting from the combined influence of founders’ psychological mechanisms and the environmental support embedded within the incubation system.

### 2.2 Business coaching and founder level of confidence

Business coaching, as a structured form of guidance and mentoring within business incubators, provides founders with reflective feedback and social learning experiences that reinforce their sense of agency and self-responsibility. According to Social Cognitive Theory ( Bandura, 2001), learning occurs in a social context through observation and interaction: founders internalize the behaviors and attributions endorsed by their coaches. Empirical and theoretical lines of evidence suggest that coaching can shift individuals’ beliefs about their control over outcomes. For instance, Ndlovu-Hlatshwayo and Msimango-Galawe (2023) highlight the critical success factors of entrepreneurial coaching in incubators, emphasizing relational aspects and coach competence as key for empowering entrepreneurs. Moreover, in adolescent educational settings, Tseng et al. (2022) find that educational interventions positively influence students’ internal locus of control, suggesting that structured developmental support can foster stronger internal control beliefs. Thus, business coaching in a university incubator context is likely to foster an internal locus of control among student-founders, by reinforcing the belief that their own actions, rather than external forces, drive business outcomes.

From a social-cognitive perspective, business coaching not only transmits knowledge but also builds self-efficacy by providing mastery experiences, verbal persuasion, and vicarious learning. According to Bandura’s theory (2001), such mechanisms are fundamental in strengthening one’s belief in one’s entrepreneurial capabilities. Empirical research demonstrates the importance of self-efficacy for startup success: Caliendo et al. (2023) show that higher self-efficacy predicts better startup survival, innovation, and income. In the context of incubation and coaching, Mohamed (2024) finds that entrepreneurial coaching positively affects the performance of nascent ventures, likely via psychological empowerment. Therefore, business coaching in university incubators can be expected to enhance founders’ self-efficacy by exposing them to guided problem-solving, feedback, and social modeling, thereby building courage, resilience, and competence in entrepreneurial tasks.

H1: Business coaching positively and significantly influences on founder locus of control

H2: Business coaching positively and significantly influences on founder self-efficacy

### 2.3 Business coaching and entrepreneurial orientation

Business coaching can exert a direct and substantial influence on a founder’s entrepreneurial orientation —a multidimensional strategic posture encompassing innovativeness, risk-taking, and proactiveness ( Azizi et al., 2023; Semivolos and Raimi, 2022). Within university business incubators, coaching serves not only as a mechanism for skill enhancement but also as a developmental intervention that shapes how founders think, behave, and interpret entrepreneurial opportunities ( Daradkeh and Mansoor, 2023; Donbesuur et al., 2020). Through iterative coaching sessions, founders internalize strategic mindsets that enable them to identify unmet market needs, experiment with novel solutions, and respond proactively to environmental changes ( Blank, 2023). Coaching provides a structured environment in which mentors and business professionals help founders engage in strategic reflection, challenge assumptions, and reframe problems—processes that are foundational in cultivating innovation-oriented behaviors ( Rosado-Cubero et al., 2024; Mohamed, 2024). Furthermore, coaching strengthens the cognitive and psychological prerequisites for entrepreneurial action. By offering constructive feedback, motivational reinforcement, and exposure to real business scenarios, coaching enhances founders’ confidence in making strategic decisions and committing resources to uncertain or high-risk ventures ( Azizi et al., 2023; Chen et al., 2023; Hamdi et al., 2023). Existing empirical research supports these mechanisms: coaching interventions have been shown to influence not only nascent firms’ operational performance but also their strategic behavior, including entrepreneurial orientation ( Daradkeh and Mansoor, 2023; Donbesuur et al., 2020; Mohamed, 2024; Rosado-Cubero et al., 2024). In the context of university incubators, structured coaching interactions thus catalyze the development of an entrepreneurial orientation by integrating experiential learning, strategic guidance, and psychological empowerment.

H3: Business coaching positively and significantly influences the founder’s entrepreneurial orientation

### 2.4 Founder level of confidence and entrepreneurial orientation

A founder’s locus of control—the extent to which they perceive outcomes as contingent on their own actions—can substantially influence their entrepreneurial orientation. Founders with a strong internal locus of control are more likely to take initiative, embrace risks, and proactively pursue innovation, as they believe they can directly impact outcomes. Empirical evidence supports this notion: Hamzah and Othman (2023) demonstrated that an internal locus of control is positively associated with entrepreneurial competency, which in turn drives business growth and promotes sustainable entrepreneurial behavior, a finding also corroborated by prior research ( Hung Kee & Rahman, 2017; Utomo et al., 2025). Although much of the existing literature has primarily focused on small business contexts ( Crompton et al., 2012; Hossain & Valliere, 2024; Leonelli et al., 2025), the exact psychological mechanisms are likely applicable to student-led startups within university incubators ( Caliendo et al., 2023; Guerrero et al., 2020). Therefore, founders exhibiting a higher internal locus of control are expected to demonstrate stronger entrepreneurial orientation, as their perception of personal agency motivates them to innovate, take calculated risks, and act proactively ( Branca et al., 2025).

In addition, self-efficacy, defined as the founder’s belief in their ability to execute entrepreneurial tasks, is theorized to positively influence entrepreneurial orientation ( Blank, 2023; Branca et al., 2025). Founders with high self-efficacy are more confident in their capacity to innovate, take risks, and act proactively, all core components of entrepreneurial orientation ( Alikhani and Shahriari, 2025). Empirical evidence supports this linkage; for instance, Newman et al. (2019) identified self-efficacy as a key antecedent of entrepreneurial behavior in a systematic review. More recently, Ye and Kang (2025) show that among college students, entrepreneurial self-efficacy significantly predicts entrepreneurial orientation and future business behavior. Also, meta-empirical studies of startups (e.g., Caliendo et al., 2023; Leitão et al., 2022) confirm that self-efficacy substantially contributes to firm survival and growth. Hence, in the university incubator context, founder self-efficacy is expected to positively affect their entrepreneurial orientation by empowering them to commit to strategic entrepreneurial behaviors ( Hossain and Valliere, 2024; Nicholls-Nixon and Maxheimer, 2022).

H4: Founder locus of control positively and significantly influences on founder entrepreneurial orientation

H5: Founder self-efficacy positively and significantly influences on founder entrepreneurial orientation

### 2.5 The mediating role

Drawing on Social Cognitive Theory, business coaching (an environmental factor) first influences a founder’s locus of control (a personal cognitive factor), which then shapes their entrepreneurial orientation (a behavioral outcome). In this mediated mechanism, coaching affects how founders attribute control, and this attribution then impacts their willingness to engage in innovation, take on risk, and be proactive. Empirical research provides support for such mediation in related contexts; for example, Hamzah and Othman (2023) found that an internal locus of control affects business outcomes through entrepreneurial competency. Similarly, the developing cognition literature suggests that coaching-style interventions can alter locus of control over time, thereby supporting strategic entrepreneurial behavior. Therefore, it is plausible that the effect of business coaching on entrepreneurial orientation is not wholly direct, but is partially or fully mediated by locus of control.

Another plausible mediating mechanism arises from Social Cognitive Theory: business coaching strengthens self-efficacy (a personal cognitive factor), which, in turn, leads to greater entrepreneurial orientation. Coaching provides mastery experiences, verbal persuasion, and social modeling that build self-efficacy, and this enhanced confidence then contributes to proactive, innovative, and risk-taking entrepreneurial behaviors. Empirical support for this claim exists in entrepreneurship research: Mohamed (2024) reports that coaching positively affects startup performance, in part through psychological empowerment. Also, self-efficacy has been shown to mediate the effect of training or support interventions on entrepreneurial behavior in educational contexts ( Setyawati & Geraldo, 2021). Thus, in the university incubator context, self-efficacy likely serves as a key mediator between business coaching and entrepreneurial orientation.

H6: Founder locus of control mediates between business coaching and entrepreneurial orientation

H7: Founder self-efficacy mediates between business coaching and entrepreneurial orientation

## 3. Method

### 3.1 Data collection and sample

**Figure 1.**
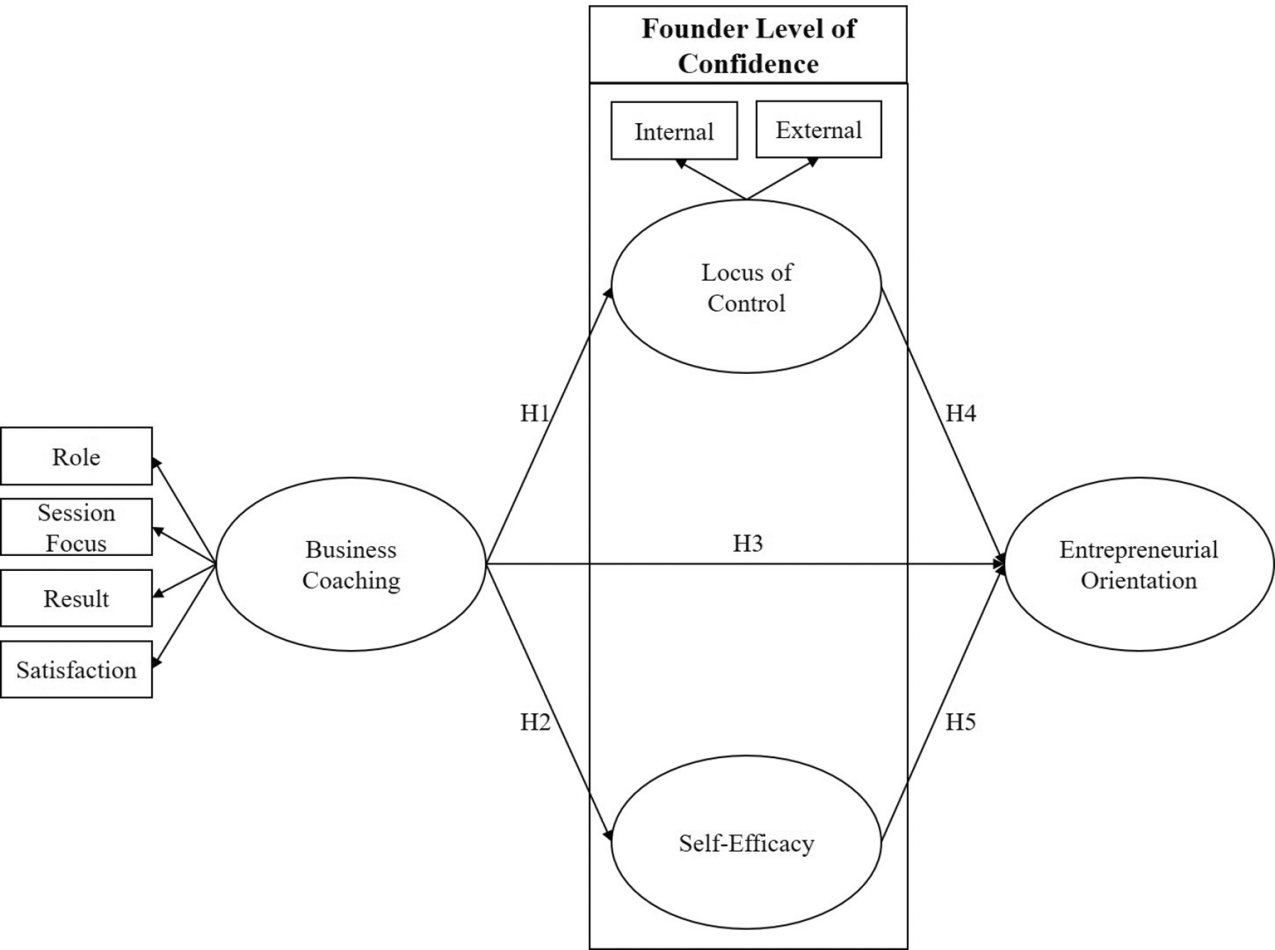
Research model.

This study employed a quantitative research approach to examine objective relationships among variables (see Figure 1), as outlined by Creswell and Creswell (2017). Data were collected using a structured online questionnaire distributed to student founders participating in university business incubator programs across Indonesia. Consequently, the participants in this study were student startup founders affiliated with university business incubators in Indonesia. All respondents were enrolled in diploma or bachelor’s degree programs and were actively developing their ventures within structured incubation environments. A purposive sampling strategy was used to ensure that only individuals who met the inclusion criteria—specifically, founders of active startups currently or recently enrolled in a university business incubator—were included in the study.

Overall, 343 questionnaires were returned; however, the final dataset included 220 valid responses, resulting in a 64.14% response rate. Since this study focuses on entrepreneurial behavior, it follows Roscoe’s (1975) recommendation of an appropriate sample size range of 30 to 500 respondents for behavioral research. Moreover, for robust estimation using Partial Least Squares Structural Equation Modeling (PLS-SEM), Hair et al. (2019) recommend a sample size of 100-400 respondents. Therefore, the sample size obtained in this study aligns with these rules of thumb, ensuring the reliability and validity of the subsequent analysis.

### 3.2 Participant and procedure

The data collection procedure began with identifying university business incubators through institutional directories, entrepreneurship centers, and national incubation networks. Permission to distribute the survey was obtained from the incubator managers, and the survey was disseminated through official communication channels. Participants were informed about the study’s purpose, assured of confidentiality, and notified that participation was voluntary. The questionnaire included screening questions to confirm founder status and current or recent involvement in an incubation program.

In compliance with ethical research standards, the introductory page provided detailed information about the study, along with an informed consent statement that asked, “Are you willing to participate as a respondent in this study?” Only respondents who provided consent were allowed to proceed to the subsequent pages. Measures to safeguard data privacy were implemented, including anonymizing responses and securely storing all collected information in accordance with relevant data protection regulations. By emphasizing these ethical considerations, the study adhered to established best practices, ensuring the protection of participants’ privacy and autonomy throughout the research process. The socio-demographic section of the questionnaire included only essential items, such as age, gender, year of startup establishment, duration of participation in the university business incubation program, industry sector, and revenue turnover. This careful selection of variables ensured the collection of relevant contextual information while minimizing respondent burden and maintaining focus on the research objectives.

### 3.3 Ethical approval and consent

This research was conducted in accordance with the ethical principles outlined in the Declaration of Helsinki (2013) for research involving human participants. Prior to the commencement of the study, ethical approval was obtained from the Ethics Committee of the Interdisciplinary School of Management Technology, Institut Teknologi Sepuluh Nopember (ITS), Indonesia. The research protocol was formally reviewed and approved under the reference number 582/IT2.IX.8/B/TU.00.09/XII/2025. The ethics review ensured that the study complied with national and international guidelines for human subject research, including procedures for informed consent, voluntary participation, confidentiality, and data protection. All participants were clearly informed about the objectives, procedures, and potential implications of the study, and written informed consent was obtained prior to their participation. Participants were assured that their involvement in the study was entirely voluntary and that they could withdraw at any stage without any consequences. To protect participants’ privacy, no personally identifiable information was collected, and all responses were treated anonymously and used solely for academic research purposes. Data handling and storage procedures adhered to applicable data protection standards, including principles aligned with the General Data Protection Regulation (GDPR).

### 3.4 Measurement

The survey instrument was developed based on the research objectives and an extensive review of prior literature. A five-point Likert scale, ranging from strongly disagree (1) to strongly agree (5), was applied to all measurement items. The measurement of constructs in this study adopted the framework proposed by Crompton et al. (2012) for business coaching, comprising four dimensions: role (2 items), session focus (3 items), results (2 items), and satisfaction (3 items). Additionally, the constructs of locus of control and self-efficacy were measured using items adapted from Crompton et al. (2012), with locus of control comprising internal (two items) and external (two items) dimensions, and self-efficacy comprising four items. For the entrepreneurial orientation construct, nine items from Wu et al. (2020) were adopted to capture its key dimensions, including innovativeness, proactiveness, and risk-taking. This structured measurement approach ensured that all constructs were operationalized with validated scales from prior empirical studies, enhancing the reliability and comparability of the findings. The consistent use of established instruments also supports the study’s methodological rigor, enabling meaningful interpretation and integration of results within the broader literature on university business incubators, founder behavior, and entrepreneurial development.

### 3.5 Data analysis

Partial Least Squares Structural Equation Modeling (PLS-SEM) is widely regarded as a robust inferential technique suitable for analyzing various types of data, as it requires relatively few distributional assumptions and can estimate relationships even when theoretical foundations are still developing ( Hair et al., 2014). A major strength of PLS-SEM lies in its capacity to estimate complex structural models and generate reliable parameter estimates when key methodological requirements are satisfied. In this study, PLS-SEM was applied to develop and test hypotheses, predict complex phenomena, and perform multivariate analysis within a quantitative framework. Its selection was primarily driven by its methodological flexibility and strong suitability for exploratory research ( Hair et al., 2016). Prior studies have also emphasized its robustness in assessing intricate theoretical frameworks involving multiple latent constructs ( Hair et al., 2022; Sarstedt et al., 2020; Hair & Alamer, 2022). The evaluation of the measurement model followed established guidelines. Internal consistency reliability was assessed using Cronbach’s alpha and composite reliability, with acceptable thresholds set above 0.70. Convergent validity was examined through indicator loadings and the average variance extracted (AVE), both of which were required to exceed 0.50. Discriminant validity was evaluated using recommended procedures ( Hair et al., 2019; 2022), particularly the heterotrait–monotrait (HTMT) ratio of correlations ( Franke & Sarstedt, 2019), ensuring that inter-construct correlations were sufficiently lower than intra-construct correlations to confirm adequate construct distinctiveness.

## 4. Result and analysis

The respondents in this study consist of 220 startup founders who are active participants in university business incubator programs across Indonesia (see Table 1). All respondents are university students, specifically those enrolled in diploma and undergraduate (bachelor’s) programs, and are simultaneously developing early-stage ventures within their respective institutional incubation ecosystems. In terms of gender distribution, the sample comprises 60.9% male and 39.1% female founders, indicating a reasonably balanced representation of student entrepreneurial participation. The respondents are generally young, reflecting the typical age range of university students: 21–23 years represent the largest proportion (43.6%), followed by those aged 24–26 years (34.5%) and 18–20 years (21.8%). Regarding startup maturity, 43.6% of the ventures have been established for 1–2 years, 30.0% for 3–4 years, and 26.4%. Participation duration in university business incubator programs also varies, with the largest proportion (36.8%) having engaged for 7–12 months, followed by 1.5 years (24.5%), less than six months (17.7%), two years (12.7%), and more than two years (8.2%). Industry representation shows that most student startups operate in technology and software (32.7%), followed by creative industries and digital content (23.6%), agriculture and agri-tech (17.3%), financial services and fintech (15.5%), and education technology (10.9%). This distribution suggests that university incubators are supporting a wide range of innovative sectors aligned with digital transformation and socio-economic development priorities. In terms of financial performance, nearly one-third of the startups (29.1%) generate less than IDR 25 million in annual revenue, while others fall within higher revenue brackets, indicating varying stages of commercial growth.

**Table 1.** Demography of respondents.

Demographic		Frequency	Percentage (%)
Gender	Male	134	60.9
	Female	86	39.1
Age	18–20 years	48	21.8
	21–23 years	96	43.6
	24–26 years	76	34.5
Startup Establishment Year	<1 year	58	26.4
	1–2 years	96	43.6
	3–4 years	66	30.0
Duration of Participation in University Business Incubation Program	<6 months	39	17.7
	7–12 months	81	36.8
	18 months (1.5 years)	54	24.5
	24 months (2 years)	28	12.7
	>24 months	18	8.2
Industry Sector	Technology & Software	72	32.7
	Creative Industries & Digital Content	52	23.6
	Financial Services & Fintech	34	15.5
	Education Technology (EdTech)	24	10.9
	Agriculture & Agri-Tech	38	17.3
Revenue Turnover	<IDR 25,000,000	64	29.1
	IDR 25,000,001 – 50,000,000	57	25.9
	IDR 50,000,001 – 75,000,000	44	20.0
	IDR 75,000,001 – 100,000,000	30	13.6
	>IDR 100,000,000	25	11.4

PLS-SEM was employed to assess the validity and reliability of the measurements, serving as the foundation for the quantitative approach (see Table 2). In this study, first-order reflective latent variables were examined for both reliability and validity at the construct and item levels. The business coaching variable was evaluated using a two-stage approach, incorporating reflective-formative higher-order constructs, whereas locus of control was assessed using a reflective-reflective higher-order construct. At the item level, this study followed the recommendations of Hair et al. (2019), which indicate that outer loadings should exceed 0.70. At the construct level, PLS-SEM evaluates reliability using composite reliability and Cronbach’s alpha, both of which are critical for confirming measurement consistency and must exceed the 0.70 threshold ( Hair et al., 2019). The results of the measurement analysis in this study indicate that all items and constructs meet these criteria, with values above 0.70. Additionally, the average variance extracted (AVE) and inter-construct correlation coefficients were calculated, showing that all constructs have AVE values exceeding 0.50. Therefore, all constructs satisfy the requirements for convergent validity ( Hair et al., 2014; 2019).

**Table 2.** Construct measurement.

Variable	Item	Outer loading	Cronbach’s alpha	Composite reliability	AVE
Business Coaching Role	BC_Role1	0.919	0.825	0.920	0.851
	BC_Role2	0.926			
Business Coaching Session Focus	BC_SesFoc1	0.887	0.891	0.933	0.822
	BC_SesFoc2	0.919			
	BC_SesFoc3	0.913			
Business Coaching Result	BC_Result1	0.895	0.727	0.880	0.785
	BC_Result2	0.877			
Business Coaching Satisfaction	BC_Satis1	0.811	0.833	0.900	0.751
	BC_Satis2	0.881			
	BC_Satis3	0.906			
Internal Locus of Control	Int_Fac1	0.939	0.876	0.942	0.890
	Int_Fac2	0.948			
External Locus of Control	Exter_Fac1	0.967	0.931	0.967	0.936
	Exter_Fac2	0.968			
Self-Efficacy	Self_Eff1	0.856	0.867	0.909	0.714
	Self_Eff2	0.866			
	Self_Eff3	0.860			
	Self_Eff4	0.797			
Entrepreneurial Orientation	EO_1	0.815	0.939	0.948	0.671
	EO_2	0.862			
	EO_3	0.836			
	EO_4	0.801			
	EO_5	0.816			
	EO_6	0.817			
	EO_7	0.837			
	EO_8	0.765			
	EO_9	0.822			

To assess discriminant validity (see Table 3), this study utilized the Heterotrait–Monotrait (HTMT) ratio of correlations, which is widely recommended for variance-based PLS-SEM analyses ( Henseler et al., 2015). Compared with the traditional Fornell-Larcker (1981) criterion, HTMT offers a more rigorous and accurate procedure because it evaluates the proportion of correlations across constructs relative to those within the same construct ( Hair & Alamer, 2022). According to the guidelines proposed by Henseler et al. (2015), discriminant validity is considered satisfactory when the HTMT value is below 0.90. All HTMT values in this research were below the 0.90 benchmark, indicating that the constructs are sufficiently distinct and do not exhibit problematic levels of conceptual overlap. This outcome confirms that each variable measures a unique theoretical dimension, thereby supporting the soundness of the measurement model. Hair and Alamer (2022) also note that HTMT is a more reliable indicator of discriminant validity within the PLS-SEM framework, as it is more sensitive to identifying validity concerns. For this reason, HTMT was selected over more traditional approaches. Ensuring adequate discriminant validity strengthens the structural model’s reliability and enhances confidence in the study’s theoretical framework.

**Table 3.** Discriminant validity.

	Entrepreneurial orientation	External	Internal	Result	Role	Satisfaction	Self-efficacy	Session focus
Entrepreneurial Orientation								
External	0.413							
Internal	0.682	0.546						
Result	0.723	0.570	0.865					
Role	0.613	0.509	0.809	0.803				
Satisfaction	0.693	0.525	0.889	0.838	0.861			
Self-Efficacy	0.594	0.298	0.620	0.802	0.653	0.709		
Session Focus	0.628	0.495	0.801	0.866	0.842	0.837	0.547	

When assessing the higher-order construct, Hair and Alamer (2022) highlight that evaluating both outer loadings and outer weights is essential for establishing construct validity. In this study, the repeated indicator approach was employed, whereby the latent variable scores of the first-order constructs were reused as indicators for the higher-order construct ( Hair and Alamer, 2022; Henseler and Chin, 2010). This technique allows each first-order dimension to be appropriately represented, ensuring that the higher-order construct accurately captures its underlying components. The path weighting scheme in SmartPLS was used to estimate relationships among constructs, and the results confirmed that each first-order construct contributed significantly to the higher-order construct. As presented in Table 4, all outer loadings and outer weights exceeded the recommended thresholds, and the outer weights were statistically significant.

**Table 4.** Outer loading and weight.

Construct	Outer loading	Outer weight
Business Coaching Role	0.872	0.221
Business Coaching Session Focus	0.868	0.269
Business Coaching Result	0.936	0.395
Business Coaching Satisfaction	0.957	0.485
Internal Locus of Control	0.939	0.424
External Locus of Control	0.780	0.720

In this study, to ensure that the analyzed data is free from bias, common method bias was assessed using the Variance Inflation Factor (VIF) values of the inner model. Based on Table 5, all VIF values in this study are less than 3.33, indicating that the model can be considered free from common method bias, as suggested by Kock (2015).

**Table 5.** Inner VIF value.

	Business coaching	Entrepreneurial orientation	Locus of control	Self-Efficacy
Business Coaching		2.220	1.000	1.000
Entrepreneurial Orientation				
Locus of Control		3.294		
Self-Efficacy		1.735		

**Figure 2.**
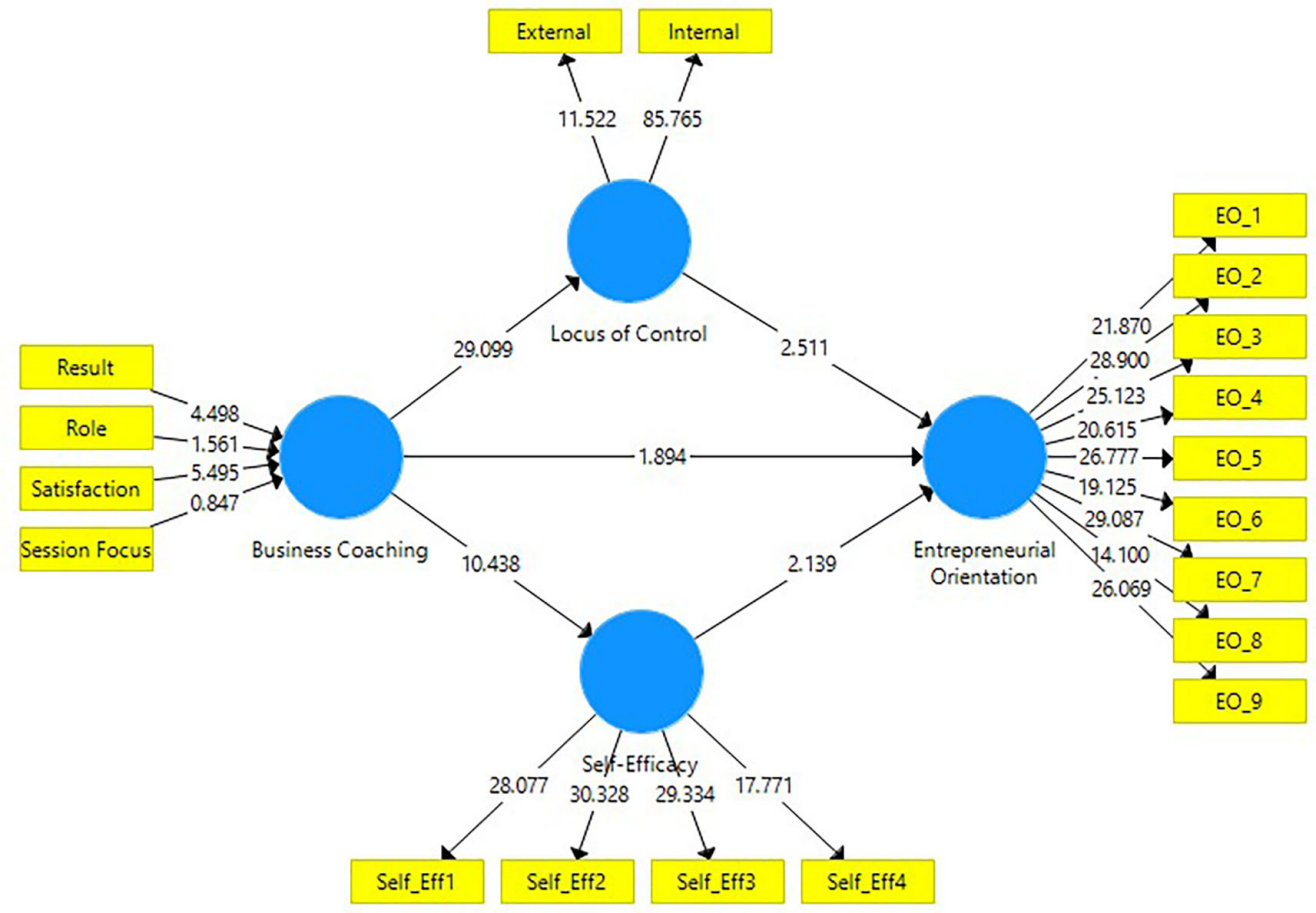
Structural model output.

At the bootstrapping stage using PLS-SEM, model fit and path coefficients were analyzed to assess the overall relationships within the model and to test the proposed hypotheses ( Figure 2). The application of a partial sequential model in the statistical analysis yielded the coefficient of determination (R ^2^), which indicates the explanatory power of the model. R ^2^ is commonly used to evaluate the strength of endogenous constructs within structural models, providing insights into their predictive relevance ( Hair et al., 2022). In the present study, the coefficient of determination (R ^2^) for locus of control (0.694); self-efficacy (0.420); and entrepreneurial orientation (0.473). These results meet the threshold level of 0.100, as suggested in prior literature (e.g. Šerić & Mikulić, 2023), confirming that the model possesses acceptable explanatory power ( Hair et al., 2017, 2022). In addition, the predictive relevance of the model was examined using the Q ^2^ statistic, which evaluates the capability of exogenous variables to predict endogenous constructs ( Hair et al., 2017). The results showed Q ^2^ values of locus of control (0.478); self-efficacy (0.289); and entrepreneurial orientation (0.303). These values exceed the recommended threshold, confirming that the structural model possesses strong predictive relevance and reflects a satisfactory level of model fit.

As presented in Table 6, this study tested the hypotheses using the bootstrapping procedure in PLS-SEM. The results indicate that business coaching has a positive and significant effect on locus of control (ß = 0.833; p < 0.05), self-efficacy (ß = 0.648; p < 0.05), and entrepreneurial orientation (ß = 0.253; p < 0.05), supporting H1, H2, and H3. Furthermore, locus of control positively and significantly influences entrepreneurial orientation (ß = 0.282; p < 0.05), and self-efficacy also positively and significantly influences entrepreneurial orientation (ß = 0.245; p < 0.05), thereby supporting H4 and H5. This study also examined the mediating roles of locus of control and self-efficacy. The results show that locus of control positively and significantly mediates the relationship between business coaching and entrepreneurial orientation (ß = 0.235; p < 0.05). Similarly, self-efficacy positively and substantially mediates the effect of business coaching on entrepreneurial orientation (ß = 0.159; p < 0.05), supporting H6 and H7. These findings demonstrate the importance of personal cognitive factors as mediators in translating business coaching into enhanced entrepreneurial orientation among student founders.

**Table 6.** Statistical effect and hypothesis testing.

Hypotheses	Direct effect (ß)	Indirect effect (ß)	T score	P values *	Conclusion
Business Coaching ➔ Locus of Control	0.833		29.099	0.000	**Accepted**
Business Coaching ➔ Self-Efficacy	0.648		10.438	0.000	**Accepted**
Business Coaching ➔ Entrepreneurial Orientation	0.253		1.894	0.039	**Accepted**
Locus of Control ➔ Entrepreneurial Orientation	0.282		2.511	0.012	**Accepted**
Self-Efficacy ➔ Entrepreneurial Orientation	0.245		2.139	0.033	**Accepted**
Business Coaching ➔ Locus of Control ➔ Entrepreneurial Orientation		0.235	2.529	0.012	**Accepted**
Business Coaching ➔ Self-Efficacy ➔ Entrepreneurial Orientation		0.159	2.125	0.034	**Accepted**

N = 220.

R ^2^ = Locus of Control (0.694); Self-Efficacy (0.420); Entrepreneurial Orientation (0.473).

* Sig. p-value < 0.05.

## 5. Discussion and implication

### 5.1 Discussion

The primary aim of this study was to examine how business coaching within university business incubators shapes the cognitive and behavioral attributes of student-founders, particularly through its effects on locus of control, self-efficacy, and entrepreneurial orientation. Grounded in Social Cognitive Theory ( Bandura, 2001), this research sought to understand the triadic interaction between environmental support (coaching), personal cognitive mechanisms (locus of control and self-efficacy), and behavioral manifestations (entrepreneurial orientation). While previous literature has explored the isolated effects of coaching or psychological traits on entrepreneurial outcomes, limited research has integrated these constructs into a holistic explanatory framework, especially in the context of university-based incubation in emerging economies. This study addresses that gap by empirically demonstrating how business coaching fosters founders’ psychological empowerment while simultaneously shaping their strategic entrepreneurial behaviors. Furthermore, the study aimed to test the mediating roles of locus of control and self-efficacy, offering insights into how and why coaching influences entrepreneurial orientation. By situating these relationships within academic incubation programs—structures that intentionally combine educational, developmental, and experiential elements—this research contributes to a deeper understanding of entrepreneurial development processes among student-founders.

The findings reveal that business coaching exerts a positive, significant influence on founders’ locus of control, self-efficacy, and entrepreneurial orientation, underscoring the central role of coaching as a developmental mechanism within university business incubators. These results are consistent with Social Cognitive Theory, which posits that individuals learn and internalize behavioral patterns through observation, guided interaction, and socially supported feedback ( Bandura, 2001). Coaching provides the reflective environment that strengthens founders’ belief that outcomes stem from their own actions—a hallmark of internal locus of control. This is aligned with Tseng et al. (2022), who showed that structured educational interventions can shift individuals toward more internal control orientations. Likewise, the coaching relationship—characterized by trust, constructive feedback, and modeling of entrepreneurial reasoning—reinforces founders’ belief in their entrepreneurial capabilities, mirroring findings by Ndlovu-Hlatshwayo and Msimango-Galawe (2023), who emphasized coach competence and relational quality as drivers of psychological empowerment in incubators. The significant influence of coaching on entrepreneurial orientation further aligns with prior findings that coaching supports not only operational decisions but also strategic thinking, innovation, and proactive behavior ( Mohamed, 2024; Daradkeh & Mansoor, 2023). Coaching effectively embeds strategic reasoning through guided reflection, scenario analysis, and iterative problem-solving, enabling founders to move beyond technical execution toward forward-looking entrepreneurial behavior.

The study also demonstrates that locus of control and self-efficacy significantly influence entrepreneurial orientation, validating these personal cognitive factors as central drivers of strategic entrepreneurial behavior. founders with an internal locus of control exhibit stronger entrepreneurial orientation because they perceive themselves as capable of influencing outcomes, leading them to engage more actively in risk-taking, innovation, and proactive opportunity-seeking. This finding aligns with Hamzah and Othman’s (2023) research, which found that an internal locus of control enhances entrepreneurial competencies that translate into sustained entrepreneurial behavior. Similarly, Leonelli et al. (2025) highlight that a strong internal belief system helps entrepreneurs navigate uncertainty by encouraging initiative and perseverance. Self-efficacy also emerged as a significant predictor of entrepreneurial orientation, reinforcing its theoretical role as a psychological mechanism that shapes an individual’s willingness to pursue challenging entrepreneurial tasks. These finding echoes evidence from Newman et al. (2019), who identify self-efficacy as a key antecedent of entrepreneurial action across contexts. Among student populations, Ye and Kang (2025) similarly demonstrate that entrepreneurial self-efficacy predicts both entrepreneurial intention and entrepreneurial orientation. In incubation settings, guided exposure to real market challenges and coach-led feedback strengthens the founders’ belief in their ability to innovate and commit resources to uncertainty ( Mohamed, 2024). Therefore, the results affirm that both locus of control and self-efficacy shape founders’ strategic posture by influencing how they interpret risks, opportunities, and their own agency in entrepreneurial environments.

The mediation results provide deeper insight into the psychological mechanisms through which business coaching influences entrepreneurial orientation. The significant mediating effect of locus of control confirms that coaching strengthens founders’ internal attributions, thereby enhancing their strategic entrepreneurial behavior. This supports SCT’s assertion that environmental cues shape personal cognition, which in turn influences behavioral outcomes ( Bandura, 2001). Coaching reframes founders’ interpretations of business challenges, shifting their explanatory style from externalized to internalized control—a cognitive shift that increases their willingness to take innovative, proactive action. This mediation is consistent with related findings by Hamzah and Othman (2023), who showed that internal locus of control acts as an intermediary mechanism linking developmental interventions and entrepreneurial competencies. Similarly, the mediating role of self-efficacy underscores how coaching enhances capability beliefs, which subsequently drive entrepreneurial orientation. By providing mastery experiences, vicarious learning, and constructive persuasion, coaching builds self-efficacy that translates into confidence to innovate and take entrepreneurial risks. Prior studies confirm this pathway: Setyawati and Geraldo (2021) found that self-efficacy mediates the effect of educational interventions on entrepreneurial behavior, while Caliendo et al. (2023) showed that higher self-efficacy contributes to opportunity recognition and startup survival. Together, the mediation results underscore that coaching does not merely provide skills—it shapes deeper cognitive structures that determine how founders behave entrepreneurially.

### 5.2 Theoretical implication

The findings of this study provide several theoretical contributions that deepen the explanatory power of Social Cognitive Theory (SCT) in the context of university-based startup incubation. First, the significant influence of business coaching on locus of control, self-efficacy, and entrepreneurial orientation extends SCT’s core propositions by demonstrating how structured environmental inputs—such as coaching, mentoring, and guided feedback—shape cognitive beliefs that subsequently drive entrepreneurial behavior ( Bandura, 2001). This aligns with emerging evidence that coaching-based interventions can alter cognitive attributions and agency beliefs among nascent entrepreneurs ( Ndlovu-Hlatshwayo & Msimango-Galawe, 2023; Tseng et al., 2022). Second, the positive effects of locus of control and self-efficacy on entrepreneurial orientation reinforce the role of personal cognitive factors as proximal predictors of strategic entrepreneurial behavior, consistent with research emphasizing psychological determinants of entrepreneurial action ( Hamzah & Othman, 2023; Newman et al., 2019; Gorgievski et al., 2023). Third, the mediation results advance theoretical understanding of how environmental and cognitive mechanisms interact. The mediation of locus of control and self-efficacy in the relationship between business coaching and entrepreneurial orientation lends empirical support to SCT’s triadic reciprocity, showing that coaching influences behavior not only directly but also through cognitive transformation ( Mohamed, 2024; Ye and Kang, 2025). These findings also align with recent arguments that entrepreneurial support programs exert their strongest impact when they modify founders’ beliefs, efficacy judgments, and perceived control ( Donbesuur et al., 2020; Rosado-Cubero et al., 2024). Collectively, this study enriches the theoretical discourse by demonstrating that business coaching functions as a psychologically embedded mechanism that shapes entrepreneurial cognition and, ultimately, strategic entrepreneurial behavior.

### 5.3 Practical implication

The findings of this study offer several actionable implications for universities, university business incubator managers, entrepreneurial coaches, and young student entrepreneurs. First, for universities, the results highlight the importance of embedding structured business coaching into broader entrepreneurial ecosystem initiatives. Since coaching significantly enhances locus of control, self-efficacy, and entrepreneurial orientation, universities should institutionalize coaching-based interventions as a core component of campus entrepreneurship programs, thereby strengthening students’ psychological and behavioral readiness to pursue high-quality venture creation. Second, for university business incubator managers, the evidence emphasizes the need to design incubation models that prioritize developmental coaching rather than merely offering technical or infrastructural support. Incubators should adopt coaching frameworks that intentionally foster internal agency, reflective learning, and entrepreneurial confidence, as these cognitive capacities have been shown to mediate strategic entrepreneurial behavior. Allocating resources toward high-quality coaching mechanisms—such as mentor matching, structured feedback cycles, and experiential learning workshops—can thus enhance incubator effectiveness.

Third, for coaches, the study underscores the essential role they play in shaping entrepreneurial cognition. Coaches should adopt approaches that combine mastery experiences, vicarious learning, and motivational reinforcement, as these elements significantly influence founders’ self-efficacy and perceived control. Professional development for coaches should be strengthened to ensure they possess both technical competence and psychological coaching skills. Finally, for young university entrepreneurs, the findings suggest that active engagement in coaching sessions can accelerate their strategic orientation, risk-taking capacity, and innovation readiness. Students should leverage coaching opportunities not merely as advisory sessions but as platforms for cognitive transformation, enabling them to develop stronger entrepreneurial mindsets and behaviors.

## 6. Conclusion, limitation, and future research

This study examined how business coaching within university business incubators influences the cognitive and strategic capacities of student founders in Indonesia, specifically through the mechanisms of locus of control, self-efficacy, and entrepreneurial orientation. Drawing on Social Cognitive Theory, the research aimed to clarify both the direct and indirect pathways through which coaching shapes entrepreneurial behavior. The empirical findings confirm that business coaching exerts a positive and significant effect on locus of control, self-efficacy, and entrepreneurial orientation. Furthermore, locus of control and self-efficacy each demonstrate substantial positive impact on entrepreneurial orientation, underscoring their roles as core cognitive drivers of strategic entrepreneurial behavior. Mediation analyses further reveal that locus of control and self-efficacy function as significant mediators in the relationship between business coaching and entrepreneurial orientation. These results collectively highlight the importance of psychologically oriented coaching interventions in shaping entrepreneurial readiness among young founders in university incubation settings.

Despite its contributions, the study is not without limitations. First, the research focuses exclusively on university business incubators in a single developing country, Indonesia. As entrepreneurial ecosystems, institutional capabilities, and incubation models differ substantially across national contexts, the findings may not be fully generalizable to business incubators operating in other regions, particularly those in developed economies. Incubators in advanced countries typically possess more mature ecosystems, stronger industry linkages, and more sophisticated support structures, which may influence the effectiveness and mechanisms of coaching in ways not captured within this study. Furthermore, cultural differences in perceptions of control, confidence, and risk-taking may alter the psychological processes examined. Thus, caution should be exercised when extending the study’s conclusions beyond similar socio-economic and institutional environments.

Given these limitations, several avenues for future research emerge. First, comparative cross-country studies—especially between developing and developed ecosystems—would enrich theoretical understanding by revealing contextual contingencies in how coaching influences entrepreneurial cognition and orientation. Second, future research could integrate additional psychological or environmental constructs, such as resilience, opportunity recognition, or institutional support quality, to expand the explanatory power of Social Cognitive Theory in entrepreneurship studies. Third, longitudinal research designs would allow scholars to examine how coaching-induced cognitive changes evolve and how they translate into long-term entrepreneurial outcomes such as venture survival, growth, or innovation performance. Finally, expanding the sampling frame to include non-university incubators or private accelerators would help bridge existing research gaps regarding how coaching functions across diverse incubation models.

## Data Availability

Zenodo. Extended Data: Research Questionnaire for The Impact of Business Coaching on Founder Confidence and Entrepreneurial Orientation in University Incubated Startups. https://doi.org/10.5281/zenodo.18059883 ( Kuswinardi et al., 2025). This project contains the following extended data:
• Research Questionnaire – the complete questionnaire instrument used in this study, consisting of structured measurement items designed to capture respondents’ perceptions, attitudes, and behavioral responses in accordance with the study’s theoretical framework. The questionnaire serves as the primary data collection instrument and supports the empirical rigor of the research. • Underlying Anonymized Dataset – respondent-level demographic and survey data used for all descriptive and inferential analyses reported in the article. The dataset is fully anonymized and de-identified to comply with the study’s ethical approval requirements and to protect participant confidentiality. No personally identifiable information is included. All data and extended materials are available under the terms of the Creative Commons Attribution 4.0 International (CC BY 4.0) license.
