# The Impact of Business Coaching on Founder Confidence and Entrepreneurial Orientation in University Incubated Startups

**DOI:** 10.12688/f1000research.175927.2

**Published:** 2026-09-12

**Authors:** Jacobus Wiwin Kuswinardi, Moses Laksono Singgih, Achmad Affandi

**Affiliations:** 1 School of Interdisciplinary Management and Technology, Institut Teknologi Sepuluh Nopember, Surabaya, East Java, Indonesia; 2 Department of Industrial and Systems Engineering, Institut Teknologi Sepuluh Nopember, Surabaya, East Java, Indonesia; 3 Electrical Engineering Department, Institut Teknologi Sepuluh Nopember, Surabaya, East Java, Indonesia

**Keywords:** Business coaching, locus of control, self-efficacy, entrepreneurial orientation, university business incubators, entrepreneurship

## Abstract

Drawing on Social Cognitive Theory, this research addresses a theoretical gap concerning *how* environmental support mechanisms in developing-country incubators foster entrepreneurial mindsets and strategic behaviour. The study examines how business coaching within university business incubators shapes the cognitive and behavioural orientations of student founders in Indonesia by analysing its influence on locus of control, self-efficacy, and entrepreneurial orientation. Throughout, *founder confidence* is used as an organizing term for two conceptually distinct agentic beliefs internal locus of control and self-efficacy each modelled as a separate mediator rather than as a single latent variable. A quantitative research design was employed, involving 220 student founders whose startups were incubated in university business incubators across Indonesia. Data were collected through structured questionnaires and analysed using PLS-SEM to test both direct and mediating effects among variables. Business coaching significantly enhances founders’ locus of control, self-efficacy, and entrepreneurial orientation. Locus of control and self-efficacy each exert positive effects on entrepreneurial orientation, confirming their roles as central cognitive mechanisms underpinning entrepreneurial behaviour. Mediation analysis reveals that locus of control and self-efficacy significantly mediate the relationship between business coaching and entrepreneurial orientation, and that the indirect cognitive pathways collectively exceed the comparatively modest direct effect of coaching. These findings position coaching not merely as a technical intervention but as a psychological and developmental process that reshapes founders’ agentic cognition and strategic entrepreneurial posture within university incubation environments. The study offers novel empirical evidence on the psychological mechanisms through which business coaching influences entrepreneurial orientation in university incubators within a developing-country context a setting often overlooked in the literature.

## 1. Introduction

In recent decades, University Business Incubators (UBIs) have emerged as key enablers that facilitate and accelerate the development of new ventures, particularly those vulnerable to uncertainty in dynamic business environments ( Wu et al., 2025). UBIs also function as an essential mechanism for strengthening the entrepreneurial university ecosystem by embedding entrepreneurship education and practice into the core functions of higher education institutions ( Etzkowitz, 2003; Longva, 2021). A well-developed entrepreneurial ecosystem within universities enhances their role not only in fostering innovation and entrepreneurial activity ( Wu et al., 2020) but also in contributing to job creation and regional economic development, rather than focusing solely on producing graduates ( Guerrero et al., 2020, 2024). The support provided by UBIs to increase the likelihood of new venture success includes technical, professional, and financial assistance ( Leitão et al., 2022), access to networked environments ( Wu et al., 2020), and structured business coaching ( Crompton et al., 2012; Kotte et al., 2021). Consequently, business incubators play a critical intermediary role in developing entrepreneurial capabilities and enhancing the resilience of startups founded by young entrepreneurs ( Branca et al., 2025; Wu et al., 2025). The capacity and capability of young founders to navigate uncertainty serve as determining factors in shaping their sense of control and emotional stability, both of which are essential for sound decision-making and long-term business sustainability ( Leonelli et al., 2025; Utomo et al., 2025).

Previous studies have highlighted that startup success rates remain exceedingly low—often below 10% ( Chakraborty et al., 2023; Krishna et al., 2016)—mainly because startups must contend with various internal risk factors, including founders’ demographic characteristics, human capital, and the quality of business planning ( Bielialov, 2022). Leonelli et al. (2025) emphasize that startups frequently overlook the behavioural complexities required to sustain business resilience. Startups also often neglect essential aspects of developing entrepreneurial orientation and founder mindset, both of which are critical determinants of success within the industry ( Hung Kee & Rahman, 2017; Utomo et al., 2025). Understanding the inherent traits of young entrepreneurs—particularly university students—remains underexplored in the entrepreneurial behaviour literature ( Utomo et al., 2025). Moreover, prior studies examining university-based new ventures in both developed and developing countries remain limited ( Mohammadi et al., 2025). Meanwhile, Crompton et al. (2012) have shown that business coaching facilitated by business incubators can enhance entrepreneurs’ self-confidence and self-efficacy. Business coaching focuses on skills development and active learning processes that help cultivate a growth mindset and self-reflection ( Kotte et al., 2021; Marvel et al., 2020; Somia et al., 2024).

Stimulating self-reflection requires the role of business coaching, which acts as an intervention within entrepreneurial practices for startups led by young entrepreneurs ( Behrendt & Greif, 2018). Business coaching emphasizes developmental interventions that leverage collaborative, reflective, and goal-oriented relationships to achieve valuable outcomes ( Behrendt & Greif, 2018; Kotte et al., 2021). Page & Holmström (2023) further highlight critical aspects of university business incubator experiences in delivering business coaching services, particularly in market research, managerial guidance, and business planning, which are essential to startup success. Crompton et al. (2012) explored the role of business coaching in enhancing entrepreneurs’ self-confidence and self-efficacy within incubator programs, demonstrating its potential to improve performance and achieve goals. The interplay among business coaching, self-confidence, and self-efficacy among young entrepreneurs has been evidenced in several studies that show strong interconnections (e.g., Arkorful & Hilton, 2022; Maxheimer & Nicholls-Nixon, 2022; Nair & Blomquist, 2021; Nicholls-Nixon & Maxheimer, 2022). Hossain and Valliere (2024) emphasize that entrepreneurial passion and self-efficacy must be actively addressed through business coaching interventions, as these factors can significantly influence startup failure rates.

Another key construct relevant to this study is entrepreneurial orientation, a firm-level strategic posture characterized by innovativeness, proactiveness, and risk-taking ( Lumpkin & Dess, 1996). Entrepreneurial orientation reflects the extent to which startups pursue innovation, anticipate market opportunities, and commit resources to uncertain ventures ( Chen et al., 2023; Daradkeh & Mansoor, 2023; Donbesuur et al., 2020). Numerous studies have established a strong link between entrepreneurial orientation and business performance across different contexts, including SMEs and startups ( Daradkeh & Mansoor, 2023; Donbesuur et al., 2020; Rosado-Cubero et al., 2024). In the specific context of university business incubators, entrepreneurial orientation refers to how founders’ strategic mindset and orientation toward entrepreneurship translate the support received from coaching into organizational behaviour and performance outcomes ( Blank, 2023). Founders with a high entrepreneurial orientation are more likely to leverage coaching to drive innovation, seize opportunities, and differentiate their ventures in competitive markets ( Rosado-Cubero et al., 2024). In incubator contexts, this translates into a greater capacity to internalize and apply external knowledge to entrepreneurial decision-making, providing strong support for the notion that university incubators do more than provide infrastructure — they actively shape nascent entrepreneurs’ strategic orientation.

Although prior work links coaching, psychological traits, and entrepreneurial outcomes, three issues remain unresolved. First, most studies estimate either coaching effects or trait effects on outcomes in isolation, leaving open whether coaching shapes strategic posture *directly*, as a behavioural transmission of skills, or *indirectly*, by restructuring the cognitive beliefs that sit at the person node of Social Cognitive Theory’s triad. Second, where mediation has been examined, the mediating cognition is typically treated as a single “confidence” variable, so it is not known whether two dissociable agentic beliefs—attributional (locus of control) and capability-based (self-efficacy)—carry the effect to different degrees. Third, these mechanisms are seldom tested among student founders in emerging-economy university incubators, a setting whose institutional immaturity and resource constraints may alter how coaching translates into cognition and behaviour. Addressing these gaps, this study models the two beliefs as parallel mediators between business coaching and entrepreneurial orientation and tests whether the direct behavioural pathway or the indirect cognitive pathways predominate. The contribution is therefore theoretical—specifying *how* coaching operates within the SCT triad—rather than merely contextual.

As an emerging economy with a growing entrepreneurial ecosystem, Indonesia has witnessed significant policy initiatives to promote university-based entrepreneurship. Programs such as the Program Pengembangan Kewirausahaan (PPK) and the Startup Innovation Program under the Ministry of Education and Culture encourage universities to develop business incubators and integrate entrepreneurship into academic curricula. Understanding how business coaching influences founders’ cognition and behaviour can inform more effective incubation strategies tailored to founders’ psychological and developmental needs.

## 2. Literature review and hypotheses development

### 2.1 Social cognitive theory

Literature on the development of student-led startups within UBIs highlights that university-based incubation plays a fundamental role in shaping the behaviours, capabilities, and strategic orientations of young entrepreneurs. UBIs provide technical, managerial, and psychosocial support, including business coaching specifically designed to strengthen reflective skills, decision-making abilities, and preparedness for uncertainty ( Kotte et al., 2021; Page & Holmström, 2023). Coaching is viewed as a developmental intervention that exposes student-founders to real business practices and encourages self-regulated learning and strategic adaptation ( Marvel et al., 2020; Somià et al., 2024). In the context of student startups—where founders often lack significant business experience—coaching serves as a catalyst that enhances cognitive, emotional, and behavioural capacities ( Arkorful & Hilton, 2022; Maxheimer & Nicholls-Nixon, 2022). Theoretically, the relationships among these variables can be explained through Social Cognitive Theory (SCT) ( Bandura, 2001). SCT posits that human behaviour is shaped by the triadic reciprocal interaction among personal factors (cognitive and affective processes), environmental influences, and behavioural outcomes. In this study, business coaching serves as an ecological determinant, providing support, feedback, and social learning opportunities. Locus of control and self-efficacy represent personal cognitive mechanisms that shape how founders process information, develop confidence in their abilities, and assess their capacity to act. Entrepreneurial orientation, in turn, is the behavioural outcome arising from the interaction between founders’ psychological conditions and the environmental support provided by UBIs.

Two psychological attributes frequently associated with the effectiveness of coaching in the incubation process are locus of control and self-efficacy. Founders with a strong internal locus of control tend to believe that their actions determine their venture’s success, making them more responsive to the learning processes facilitated by coaches and incubators ( Newman et al., 2019). Self-efficacy has been consistently shown to influence risk-taking, opportunity evaluation, and resilience in the face of failure ( Baron & Hmieleski, 2018; Leonelli et al., 2025). UBIs play an essential role in strengthening self-efficacy by providing mentoring, business simulations, and exposure to supportive entrepreneurial environments ( Nair & Blomquist, 2021; Rosado-Cubero et al., 2024). Both characteristics shape the extent to which founders can effectively utilize incubation resources, including networking opportunities and business model validation. Beyond individual psychological factors, the literature underscores the importance of entrepreneurial orientation (EO) as a determinant of student startup success. EO—reflecting innovativeness, proactiveness, and risk-taking—has been widely recognized as a key antecedent of startup performance ( Chen et al., 2023; Daradkeh & Mansoor, 2023). Within university environments, EO is influenced by the interaction between founder attributes and incubation support ( Rosado-Cubero et al., 2024). UBIs strengthen EO through knowledge transfer, coaching interventions, and access to professional networks ( Blank, 2023). EO can therefore be viewed as a behavioural manifestation resulting from the combined influence of founders’ psychological mechanisms and the environmental support embedded within the incubation system.

### 2.2 Business coaching and founder level of confidence

In this study, *founder confidence* is used as an organizing term rather than as a single measured variable. It refers to the agentic belief system through which student founders judge their relationship to entrepreneurial outcomes, and it comprises two conceptually distinct components that we model as separate constructs: (i) internal locus of control, an attributional belief concerning whether venture outcomes are contingent on the founder’s own actions rather than on external forces; and (ii) entrepreneurial self-efficacy, a capability belief concerning the founder’s confidence in performing specific entrepreneurial tasks. These components are related but not interchangeable: locus of control addresses *who or what causes* outcomes, whereas self-efficacy addresses *whether one can execute* the actions that produce them. We therefore do not treat founder confidence as a statistical higher-order latent variable; instead, the two beliefs enter the model independently, allowing their distinct mediating roles to be estimated. Throughout the paper, “entrepreneurial cognition” denotes the broader category of founder cognitive-motivational processes and is not used as a synonym for either measured construct.

Business coaching, as a structured form of guidance and mentoring within business incubators, provides founders with reflective feedback and social learning experiences that reinforce their sense of agency and self-responsibility. According to SCT ( Bandura, 2001), learning occurs in a social context through observation and interaction: founders internalize the behaviours and attributions endorsed by their coaches. Empirical and theoretical lines of evidence suggest that coaching can shift individuals’ beliefs about their control over outcomes. Ndlovu-Hlatshwayo & Msimango-Galawe (2023) highlight the critical success factors of entrepreneurial coaching in incubators, emphasizing relational aspects and coach competence as key for empowering entrepreneurs. In adolescent educational settings, Tseng et al. (2022) find that educational interventions positively influence students’ internal locus of control, suggesting that structured developmental support can foster stronger internal control beliefs. Business coaching in a university incubator context is thus likely to foster an internal locus of control among student-founders by reinforcing the belief that their own actions, rather than external forces, drive business outcomes.

From a social-cognitive perspective, business coaching not only transmits knowledge but also builds self-efficacy by providing mastery experiences, verbal persuasion, and vicarious learning. According to Bandura’s theory (2001), such mechanisms are fundamental in strengthening one’s belief in one’s entrepreneurial capabilities. Caliendo et al. (2023) show that higher self-efficacy predicts better startup survival, innovation, and income. In the context of incubation and coaching, Mohamed (2024) finds that entrepreneurial coaching positively affects the performance of nascent ventures, likely via psychological empowerment. Business coaching in university incubators can therefore be expected to enhance founders’ self-efficacy by exposing them to guided problem-solving, feedback, and social modelling, thereby building courage, resilience, and competence in entrepreneurial tasks.

H1: Business coaching positively and significantly influences on founder locus of control

H2: Business coaching positively and significantly influences on founder self-efficacy

### 2.3 Business coaching and entrepreneurial orientation

Business coaching can exert a direct influence on a founder’s entrepreneurial orientation —a multidimensional strategic posture encompassing innovativeness, risk-taking, and proactiveness ( Azizi et al., 2023; Semivolos & Raimi, 2022). Within UBIs, coaching serves not only as a mechanism for skill enhancement but also as a developmental intervention that shapes how founders think, behave, and interpret entrepreneurial opportunities ( Daradkeh & Mansoor, 2023; Donbesuur et al., 2020). Through iterative coaching sessions, founders internalize strategic mindsets that enable them to identify unmet market needs, experiment with novel solutions, and respond proactively to environmental changes ( Blank, 2023). Coaching provides a structured environment in which mentors and business professionals help founders engage in strategic reflection, challenge assumptions, and reframe problems—processes foundational in cultivating innovation-oriented behaviours ( Rosado-Cubero et al., 2024; Mohamed, 2024). By offering constructive feedback, motivational reinforcement, and exposure to real business scenarios, coaching enhances founders’ confidence in making strategic decisions and committing resources to uncertain or high-risk ventures ( Azizi et al., 2023; Chen et al., 2023; Hamdi et al., 2023). Existing empirical research supports these mechanisms: coaching interventions have been shown to influence not only nascent firms’ operational performance but also their strategic behaviour, including entrepreneurial orientation ( Daradkeh & Mansoor, 2023; Donbesuur et al., 2020; Mohamed, 2024; Rosado-Cubero et al., 2024). In university incubators, structured coaching interactions thus catalyze the development of an entrepreneurial orientation by integrating experiential learning, strategic guidance, and psychological empowerment.

H3: Business coaching positively and significantly influences the founder’s entrepreneurial orientation

### 2.4 Founder level of confidence and entrepreneurial orientation

A founder’s locus of control—the extent to which they perceive outcomes as contingent on their own actions—can substantially influence their entrepreneurial orientation. Founders with a strong internal locus of control are more likely to take initiative, embrace risks, and proactively pursue innovation, as they believe they can directly impact outcomes. Hamzah & Othman (2023) demonstrated that an internal locus of control is positively associated with entrepreneurial competency, which in turn drives business growth and promotes sustainable entrepreneurial behaviour, a finding corroborated by prior research ( Hung Kee & Rahman, 2017; Utomo et al., 2025). Although much of the existing literature has primarily focused on small business contexts ( Crompton et al., 2012; Hossain & Valliere, 2024; Leonelli et al., 2025), the same psychological mechanisms are likely applicable to student-led startups within university incubators ( Caliendo et al., 2023; Guerrero et al., 2020). Founders exhibiting a higher internal locus of control are therefore expected to demonstrate stronger entrepreneurial orientation, as their perception of personal agency motivates them to innovate, take calculated risks, and act proactively ( Branca et al., 2025).

Self-efficacy, defined as the founder’s belief in their ability to execute entrepreneurial tasks, is theorized to positively influence entrepreneurial orientation ( Blank, 2023; Branca et al., 2025). Founders with high self-efficacy are more confident in their capacity to innovate, take risks, and act proactively—core components of entrepreneurial orientation ( Alikhani & Shahriari, 2025). Newman et al. (2019) identified self-efficacy as a key antecedent of entrepreneurial behaviour in a systematic review and Ye and Kang (2025) show that among college students, entrepreneurial self-efficacy significantly predicts entrepreneurial orientation and future business behaviour. Meta-empirical studies of startups (e.g., Caliendo et al., 2023; Leitão et al., 2022) confirm that self-efficacy substantially contributes to firm survival and growth. In the university incubator context, founder self-efficacy is thus expected to positively affect entrepreneurial orientation by empowering founders to commit to strategic entrepreneurial behaviours ( Hossain & Valliere, 2024; Nicholls-Nixon & Maxheimer, 2022).

H4: Founder locus of control positively and significantly influences on founder entrepreneurial orientation

H5: Founder self-efficacy positively and significantly influences on founder entrepreneurial orientation

### 2.5 The mediating role

Drawing on SCT, business coaching (an environmental factor) first influences a founder’s locus of control (a personal cognitive factor), which then shapes their entrepreneurial orientation (a behavioural outcome). In this mediated mechanism, coaching affects how founders attribute control, and this attribution then impacts their willingness to engage in innovation, take on risk, and be proactive. Hamzah and Othman (2023) found that an internal locus of control affects business outcomes through entrepreneurial competency; similarly, the developing-cognition literature suggests that coaching-style interventions can alter locus of control over time, thereby supporting strategic entrepreneurial behaviour. It is therefore plausible that the effect of business coaching on entrepreneurial orientation is not wholly direct but is partially or fully mediated by locus of control.

A second plausible mediating mechanism arises from SCT: business coaching strengthens self-efficacy (a personal cognitive factor), which in turn, leads to greater entrepreneurial orientation. Coaching provides mastery experiences, verbal persuasion, and social modelling that build self-efficacy, and this enhanced confidence then contributes to proactive, innovative, and risk-taking entrepreneurial behaviours. Mohamed (2024) reports that coaching positively affects startup performance, in part through psychological empowerment, and self-efficacy has been shown to mediate the effect of training or support interventions on entrepreneurial behaviour in educational contexts ( Setyawati & Geraldo, 2021). In the university incubator context, self-efficacy likely serves as a key mediator between business coaching and entrepreneurial orientation.

H6: Founder locus of control mediates between business coaching and entrepreneurial orientation

H7: Founder self-efficacy mediates between business coaching and entrepreneurial orientation

## 3. Method

### 3.1 Data collection and sample

**Figure 1.**
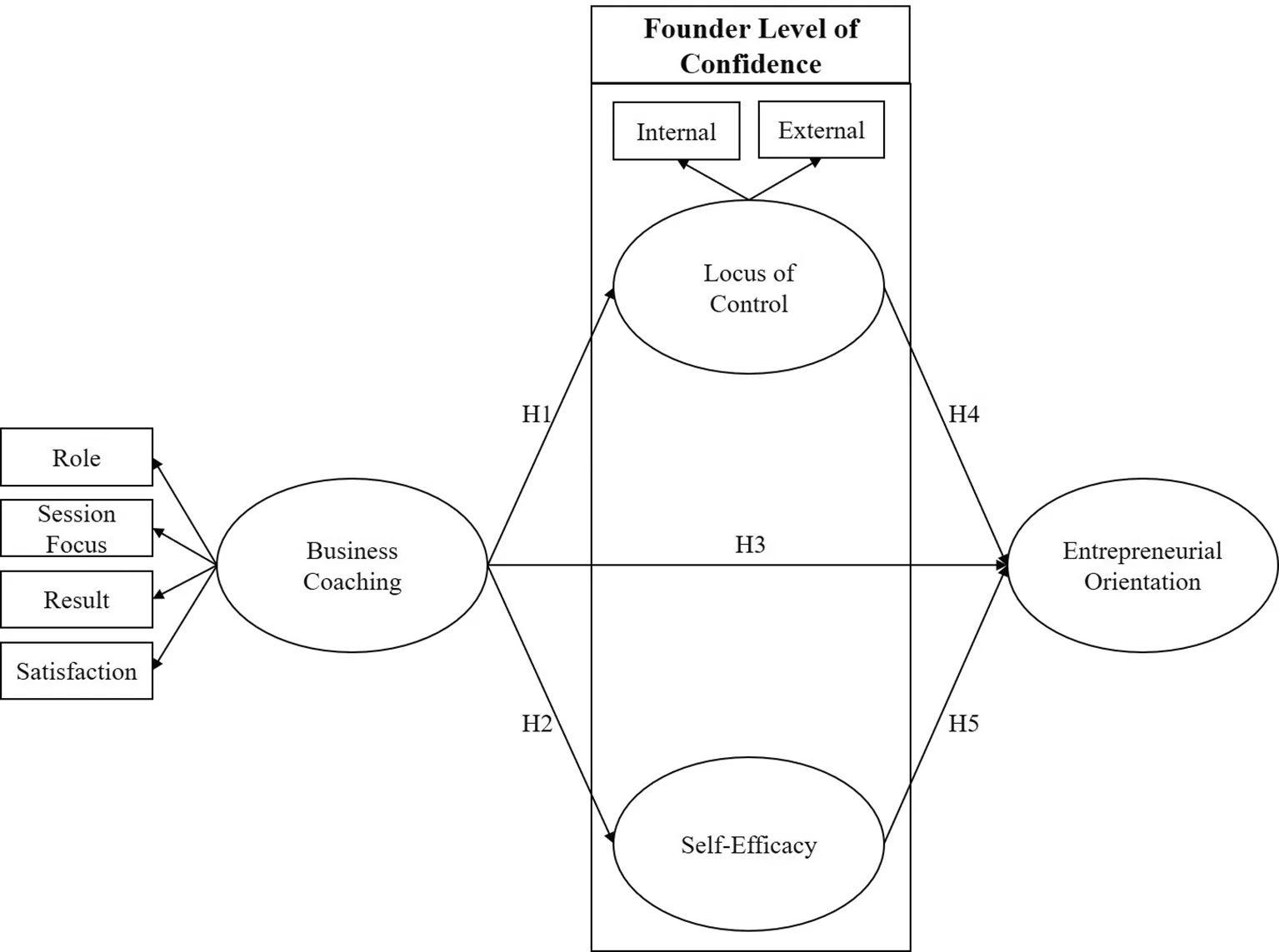
Research model.

This study employed a quantitative research approach to examine objective relationships among variables (see Figure 1), as outlined by Creswell and Creswell (2017). Data were collected using a structured online questionnaire distributed to student founders participating in university business incubator programs across Indonesia. The participants were student startup founders affiliated with university business incubators in Indonesia. All respondents were enrolled in diploma or bachelor’s degree programs and were actively developing their ventures within structured incubation environments. A purposive sampling strategy was used to ensure that only individuals meeting the inclusion criteria—founders of active startups currently or recently enrolled in a university business incubator—were included.

Overall, 343 questionnaires were returned; however, the final dataset included 220 valid responses, resulting in a 64.14% response rate. Since this study focuses on entrepreneurial behaviour, it follows Roscoe’s (1975) recommendation of an appropriate sample size range of 30 to 500 respondents for behavioural research. Moreover, for robust estimation using Partial Least Squares Structural Equation Modeling (PLS-SEM), Hair et al. (2019) recommend a sample size of 100-400 respondents. Therefore, the sample size obtained in this study aligns with these rules of thumb, ensuring the reliability and validity of the subsequent analysis.

### 3.2 Participant and procedure

The data collection procedure began with identifying university business incubators through institutional directories, entrepreneurship centers, and national incubation networks. Permission to distribute the survey was obtained from the incubator managers, and the survey was disseminated through official communication channels. Participants were informed about the study’s purpose, assured of confidentiality, and notified that participation was voluntary. The questionnaire included screening questions to confirm founder status and current or recent involvement in an incubation program.

In compliance with ethical research standards, the introductory page provided detailed information about the study, along with an informed consent statement that asked, “Are you willing to participate as a respondent in this study?” Only respondents who provided consent were allowed to proceed to the subsequent pages. Measures to safeguard data privacy were implemented, including anonymizing responses and securely storing all collected information in accordance with relevant data protection regulations. By emphasizing these ethical considerations, the study adhered to established best practices, ensuring the protection of participants’ privacy and autonomy throughout the research process. The socio-demographic section of the questionnaire included only essential items, such as age, gender, year of startup establishment, duration of participation in the university business incubation program, industry sector, and revenue turnover. This careful selection of variables ensured the collection of relevant contextual information while minimizing respondent burden and maintaining focus on the research objectives.

### 3.3 Ethical approval and consent

This research was conducted in accordance with the ethical principles outlined in the Declaration of Helsinki (2013) for research involving human participants. Prior to the commencement of the study, ethical approval was obtained from the Ethics Committee of the Interdisciplinary School of Management Technology, Institut Teknologi Sepuluh Nopember (ITS), Indonesia. The research protocol was formally reviewed and approved under the reference number 582/IT2.IX.8/B/TU.00.09/XII/2025. The ethics review ensured that the study complied with national and international guidelines for human subject research, including procedures for informed consent, voluntary participation, confidentiality, and data protection. All participants were clearly informed about the objectives, procedures, and potential implications of the study, and written informed consent was obtained prior to their participation. Participants were assured that their involvement in the study was entirely voluntary and that they could withdraw at any stage without any consequences. To protect participants’ privacy, no personally identifiable information was collected, and all responses were treated anonymously and used solely for academic research purposes. Data handling and storage procedures adhered to applicable data protection standards, including principles aligned with the General Data Protection Regulation (GDPR).

### 3.4 Measurement

The survey instrument was developed based on the research objectives and an extensive review of prior literature. A five-point Likert scale (1 = strongly disagree to 5 = strongly agree) was applied to all items. Business coaching adopted the framework of Crompton et al. (2012), comprising four dimensions: role (2 items), session focus (3 items), results (2 items), and satisfaction (3 items). Locus of control and self-efficacy used items adapted from Crompton et al. (2012), with locus of control comprising internal (2 items) and external (2 items) dimensions and self-efficacy comprising four items. For entrepreneurial orientation, nine items from Wu et al. (2020) captured innovativeness, proactiveness, and risk-taking.

Each construct was operationalized to capture the specific belief or behaviour it represents. Business coaching was measured through four experience-based facets—role clarity, session focus, results, and satisfaction—because these capture what founders actually receive and perceive from coaching rather than coach attributes alone, which suits an incubation setting where coaching is delivered as a structured service. Locus of control used internal and external items to reflect its attributional nature. Self-efficacy items referred to belief in executing entrepreneurial tasks, consistent with a capability construct. Entrepreneurial orientation used innovativeness, proactiveness, and risk-taking items to capture strategic posture as a behavioural outcome. This mapping aligns each indicator set with the SCT element it represents—environmental input, personal cognition, and behavioural outcome, respectively—maximizing content and construct validity.

Instrument adaptation followed established cross-cultural procedures. The original English items were translated into Indonesian and independently back-translated to verify semantic equivalence confirm whether translation/back-translation was performed and by whom. Content validity was assessed by number subject-matter experts, and a pilot test with number student founders was conducted prior to full-scale collection; report pilot reliability, e.g., Cronbach’s alpha ≥0.70, and any items revised.

### 3.5 Data analysis

PLS-SEM is a robust inferential technique suitable for analysing various types of data, as it requires relatively few distributional assumptions and can estimate relationships even when theoretical foundations are still developing ( Hair et al., 2014). PLS-SEM was used to develop and test hypotheses, predict complex phenomena, and perform multivariate analysis within a quantitative framework; its selection was driven by its methodological flexibility and suitability for exploratory, complex models with multiple latent constructs ( Hair et al., 2016, 2022; Sarstedt et al., 2020; Hair & Alamer, 2022). Internal consistency reliability was assessed using Cronbach’s alpha and composite reliability (thresholds > 0.70). Convergent validity was examined through indicator loadings and average variance extracted (AVE > 0.50). Discriminant validity was evaluated using the heterotrait–monotrait (HTMT) ratio of correlations ( Franke & Sarstedt, 2019; Henseler et al., 2015).

The model was estimated in SmartPLS 4 using the path-weighting scheme. Business coaching was modelled as a reflective-formative higher-order construct and locus of control as a reflective-reflective higher-order construct, using the two-stage/repeated-indicator approach ( Hair & Alamer, 2022; Henseler & Chin, 2010). Significance was assessed with bootstrapping using 10,000 subsamples and bias-corrected and accelerated (BCa) confidence intervals. Because all hypotheses are directional, one-tailed tests were applied at α = 0.05. Mediation was tested following Sarstedt et al. (2020) by examining the significance of indirect effects and their bootstrap confidence intervals, with the direct effect retained to characterize the type of mediation (partial vs full).

Common method bias was assessed in three ways. First, a full-collinearity test of the inner model showed all VIF values below the 3.33 threshold ( Kock, 2015); the highest value, locus of control → entrepreneurial orientation (VIF = 3.294), approaches but does not exceed the threshold and is reported transparently. Second, Harman’s single-factor test indicated that the first unrotated factor explained 35% of the variance. Third, the low inter-construct HTMT values (all < 0.90) provided converging evidence that common method variance is unlikely to bias the estimates. Non-response bias was evaluated by comparing early and late respondents (first vs last 25% to reply) on the main constructs using independent-samples t-tests; no significant differences emerged (all p > 0.05).

## 4. Result and analysis

The respondents in this study consist of 220 startup founders who are active participants in university business incubator programs across Indonesia (see Table 1). All respondents are university students, specifically those enrolled in diploma and undergraduate (bachelor’s) programs, and are simultaneously developing early-stage ventures within their respective institutional incubation ecosystems. In terms of gender distribution, the sample comprises 60.9% male and 39.1% female founders, indicating a reasonably balanced representation of student entrepreneurial participation. The respondents are generally young, reflecting the typical age range of university students: 21–23 years represent the largest proportion (43.6%), followed by those aged 24–26 years (34.5%) and 18–20 years (21.8%). Regarding startup maturity, 43.6% of the ventures have been established for 1–2 years, 30.0% for 3–4 years, and 26.4%. Participation duration in university business incubator programs also varies, with the largest proportion (36.8%) having engaged for 7–12 months, followed by 1.5 years (24.5%), less than six months (17.7%), two years (12.7%), and more than two years (8.2%). Industry representation shows that most student startups operate in technology and software (32.7%), followed by creative industries and digital content (23.6%), agriculture and agri-tech (17.3%), financial services and fintech (15.5%), and education technology (10.9%). This distribution suggests that university incubators are supporting a wide range of innovative sectors aligned with digital transformation and socio-economic development priorities. In terms of financial performance, nearly one-third of the startups (29.1%) generate less than IDR 25 million in annual revenue, while others fall within higher revenue brackets, indicating varying stages of commercial growth.

**Table 1.** Demography of respondents.

Demographic		Frequency	Percentage (%)
Gender	Male	134	60.9
	Female	86	39.1
Age	18–20 years	48	21.8
	21–23 years	96	43.6
	24–26 years	76	34.5
Startup Establishment Year	<1 year	58	26.4
	1–2 years	96	43.6
	3–4 years	66	30.0
Duration of Participation in University Business Incubation Program	<6 months	39	17.7
	7–12 months	81	36.8
	18 months (1.5 years)	54	24.5
	24 months (2 years)	28	12.7
	>24 months	18	8.2
Industry Sector	Technology & Software	72	32.7
	Creative Industries & Digital Content	52	23.6
	Financial Services & Fintech	34	15.5
	Education Technology (EdTech)	24	10.9
	Agriculture & Agri-Tech	38	17.3
Revenue Turnover	<IDR 25,000,000	64	29.1
	IDR 25,000,001 – 50,000,000	57	25.9
	IDR 50,000,001 – 75,000,000	44	20.0
	IDR 75,000,001 – 100,000,000	30	13.6
	>IDR 100,000,000	25	11.4

PLS-SEM was employed to assess the validity and reliability of the measurements, serving as the foundation for the quantitative approach (see Table 2). In this study, first-order reflective latent variables were examined for both reliability and validity at the construct and item levels. The business coaching variable was evaluated using a two-stage approach, incorporating reflective-formative higher-order constructs, whereas locus of control was assessed using a reflective-reflective higher-order construct. At the item level, this study followed the recommendations of Hair et al. (2019), which indicate that outer loadings should exceed 0.70. At the construct level, PLS-SEM evaluates reliability using composite reliability and Cronbach’s alpha, both of which are critical for confirming measurement consistency and must exceed the 0.70 threshold ( Hair et al., 2019). The results of the measurement analysis in this study indicate that all items and constructs meet these criteria, with values above 0.70. Additionally, the average variance extracted (AVE) and inter-construct correlation coefficients were calculated, showing that all constructs have AVE values exceeding 0.50. Therefore, all constructs satisfy the requirements for convergent validity ( Hair et al., 2014, 2019).

**Table 2.** Construct measurement.

Variable	Item	Outer loading	Cronbach’s alpha	Composite reliability	AVE
Business Coaching Role	BC_Role1	0.919	0.825	0.920	0.851
	BC_Role2	0.926			
Business Coaching Session Focus	BC_SesFoc1	0.887	0.891	0.933	0.822
	BC_SesFoc2	0.919			
	BC_SesFoc3	0.913			
Business Coaching Result	BC_Result1	0.895	0.727	0.880	0.785
	BC_Result2	0.877			
Business Coaching Satisfaction	BC_Satis1	0.811	0.833	0.900	0.751
	BC_Satis2	0.881			
	BC_Satis3	0.906			
Internal Locus of Control	Int_Fac1	0.939	0.876	0.942	0.890
	Int_Fac2	0.948			
External Locus of Control	Exter_Fac1	0.967	0.931	0.967	0.936
	Exter_Fac2	0.968			
Self-Efficacy	Self_Eff1	0.856	0.867	0.909	0.714
	Self_Eff2	0.866			
	Self_Eff3	0.860			
	Self_Eff4	0.797			
Entrepreneurial Orientation	EO_1	0.815	0.939	0.948	0.671
	EO_2	0.862			
	EO_3	0.836			
	EO_4	0.801			
	EO_5	0.816			
	EO_6	0.817			
	EO_7	0.837			
	EO_8	0.765			
	EO_9	0.822			

To assess discriminant validity (see Table 3), this study utilized the Heterotrait–Monotrait (HTMT) ratio of correlations, which is widely recommended for variance-based PLS-SEM analyses ( Henseler et al., 2015). Compared with the traditional Fornell-Larcker (1981) criterion, HTMT offers a more rigorous and accurate procedure because it evaluates the proportion of correlations across constructs relative to those within the same construct ( Hair & Alamer, 2022). According to the guidelines proposed by Henseler et al. (2015), discriminant validity is considered satisfactory when the HTMT value is below 0.90. All HTMT values in this research were below the 0.90 benchmark, indicating that the constructs are sufficiently distinct and do not exhibit problematic levels of conceptual overlap. This outcome confirms that each variable measures a unique theoretical dimension, thereby supporting the soundness of the measurement model. Hair & Alamer (2022) also note that HTMT is a more reliable indicator of discriminant validity within the PLS-SEM framework, as it is more sensitive to identifying validity concerns. For this reason, HTMT was selected over more traditional approaches. Ensuring adequate discriminant validity strengthens the structural model’s reliability and enhances confidence in the study’s theoretical framework.

**Table 3.** Discriminant validity.

	Entrepreneurial orientation	External	Internal	Result	Role	Satisfaction	Self-efficacy	Session focus
Entrepreneurial Orientation								
External	0.413							
Internal	0.682	0.546						
Result	0.723	0.570	0.865					
Role	0.613	0.509	0.809	0.803				
Satisfaction	0.693	0.525	0.889	0.838	0.861			
Self-Efficacy	0.594	0.298	0.620	0.802	0.653	0.709		
Session Focus	0.628	0.495	0.801	0.866	0.842	0.837	0.547	

When assessing the higher-order construct, Hair and Alamer (2022) highlight that evaluating both outer loadings and outer weights is essential for establishing construct validity. In this study, the repeated indicator approach was employed, whereby the latent variable scores of the first-order constructs were reused as indicators for the higher-order construct ( Hair & Alamer, 2022; Henseler & Chin, 2010). This technique allows each first-order dimension to be appropriately represented, ensuring that the higher-order construct accurately captures its underlying components. The path weighting scheme in SmartPLS was used to estimate relationships among constructs, and the results confirmed that each first-order construct contributed significantly to the higher-order construct. As presented in Table 4, all outer loadings and outer weights exceeded the recommended thresholds, and the outer weights were statistically significant.

**Table 4.** Outer loading and weight.

Construct	Outer loading	Outer weight
Business Coaching Role	0.872	0.221
Business Coaching Session Focus	0.868	0.269
Business Coaching Result	0.936	0.395
Business Coaching Satisfaction	0.957	0.485
Internal Locus of Control	0.939	0.424
External Locus of Control	0.780	0.720

Common method bias was assessed using the inner-model VIF values ( Table 5). All values are below 3.33, indicating the model can be considered free from serious common method bias ( Kock, 2015). The highest value (locus of control → entrepreneurial orientation, 3.294) is reported transparently and, together with Harman’s single-factor test and the low HTMT values, does not indicate a problematic level of common method variance.

**Table 5.** Inner VIF value.

	Business coaching	Entrepreneurial orientation	Locus of control	Self-Efficacy
Business Coaching		2.220	1.000	1.000
Entrepreneurial Orientation				
Locus of Control		3.294		
Self-Efficacy		1.735		

**Figure 2.**
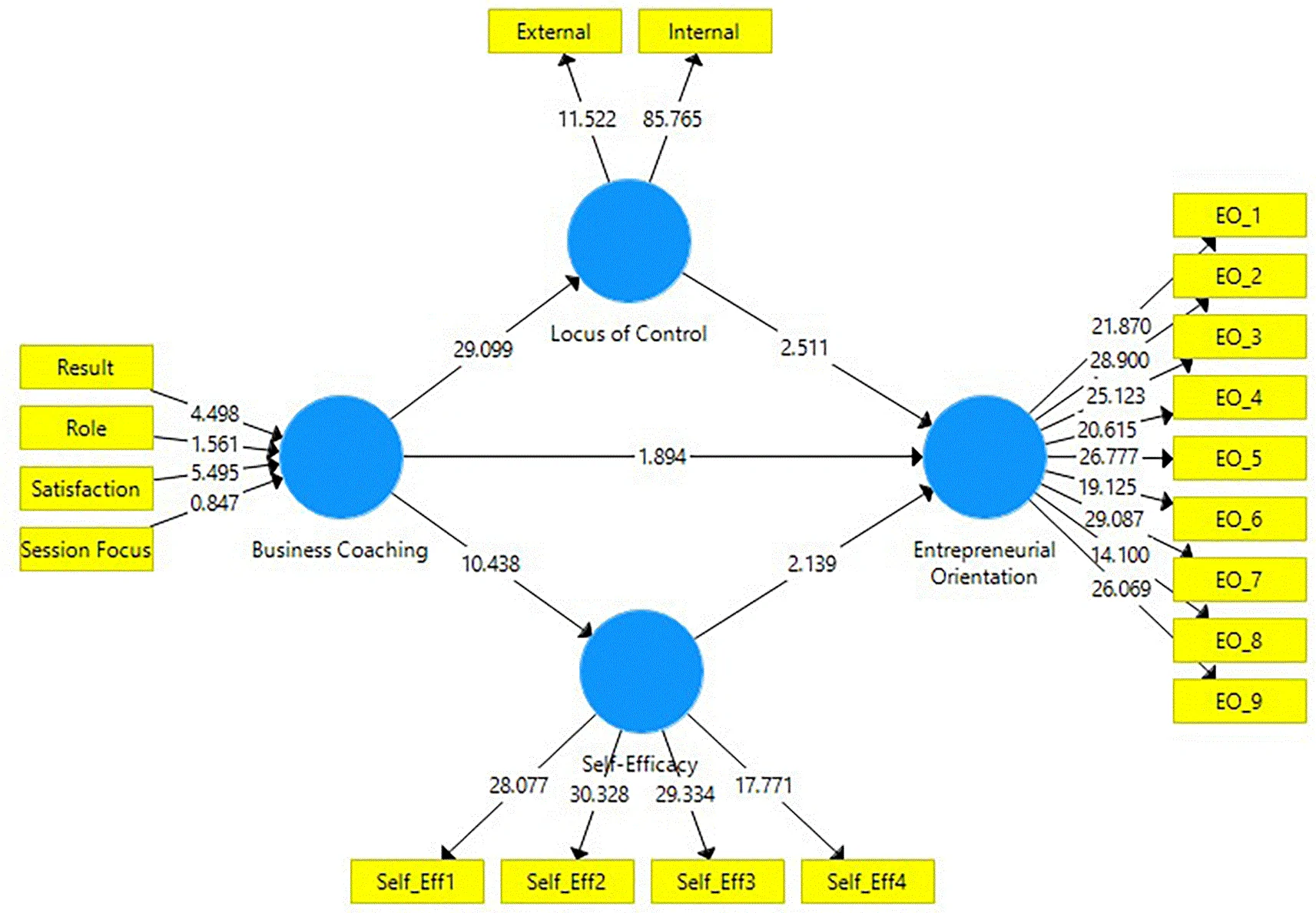
Structural model output.

At the bootstrapping stage, model fit and path coefficients were analysed ( Figure 2). The coefficient of determination (R ^2^) was 0.694 for locus of control, 0.420 for self-efficacy, and 0.473 for entrepreneurial orientation, exceeding the 0.100 threshold ( Šerić & Mikulić, 2023) and confirming acceptable explanatory power ( Hair et al., 2017, 2022). Predictive relevance (Q ^2^) was 0.478 for locus of control, 0.289 for self-efficacy, and 0.303 for entrepreneurial orientation, exceeding the recommended threshold and confirming satisfactory predictive relevance.

Beyond significance, we assessed effect sizes (f ^2^) and out-of-sample predictive power (PLSpredict). Effect sizes are reported in Table 6a. The PLSpredict procedure (k = 10 folds, 10 repetitions) indicated that the majority of/all indicators of entrepreneurial orientation yielded lower prediction error under the PLS model than the naïve linear-model benchmark (Q ^2^predict > 0), supporting the model’s predictive relevance.

**Table 6.** Statistical effect and hypothesis testing.

Hypotheses	Direct effect (β)	Indirect effect (β)	T score	P values *	Conclusion
Business Coaching ➔ Locus of Control	0.833		29.099	0.000	**Accepted**
Business Coaching ➔ Self-Efficacy	0.648		10.438	0.000	**Accepted**
Business Coaching ➔ Entrepreneurial Orientation	0.253		1.894	0.039	**Accepted**
Locus of Control ➔ Entrepreneurial Orientation	0.282		2.511	0.012	**Accepted**
Self-Efficacy ➔ Entrepreneurial Orientation	0.245		2.139	0.033	**Accepted**
Business Coaching ➔ Locus of Control ➔ Entrepreneurial Orientation		0.235	2.529	0.012	**Accepted**
Business Coaching ➔ Self-Efficacy ➔ Entrepreneurial Orientation		0.159	2.125	0.034	**Accepted**

N = 220.

R ^2^ = Locus of Control (0.694); Self-Efficacy (0.420); Entrepreneurial Orientation (0.473).

* Sig. p-value < 0.05.

As presented in Table 6, this study tested the hypotheses using the bootstrapping procedure in PLS-SEM. The results indicate that business coaching has a positive and significant effect on locus of control (β = 0.833; p < 0.05), self-efficacy (β = 0.648; p < 0.05), and entrepreneurial orientation (β = 0.253; p < 0.05), supporting H1, H2, and H3. Furthermore, locus of control positively and significantly influences entrepreneurial orientation (β = 0.282; p < 0.05), and self-efficacy also positively and significantly influences entrepreneurial orientation (β = 0.245; p < 0.05), thereby supporting H4 and H5. This study also examined the mediating roles of locus of control and self-efficacy. The results show that locus of control positively and significantly mediates the relationship between business coaching and entrepreneurial orientation (β = 0.235; p < 0.05). Similarly, self-efficacy positively and substantially mediates the effect of business coaching on entrepreneurial orientation (β = 0.159; p < 0.05), supporting H6 and H7. These findings demonstrate the importance of personal cognitive factors as mediators in translating business coaching into enhanced entrepreneurial orientation among student founders.

## 5. Discussion and implication

### 5.1 Discussion

The primary aim of this study was to examine how business coaching within university business incubators shapes the cognitive and behavioural attributes of student-founders, particularly through its effects on locus of control, self-efficacy, and entrepreneurial orientation. Read against the SCT triad, the results locate coaching’s influence primarily at the *person* node: business coaching (environmental input) most strongly predicted the two cognitive beliefs (β = 0.833 for locus of control; β = 0.648 for self-efficacy), whereas its direct link to the behavioural outcome, entrepreneurial orientation, was weaker (β = 0.253). Both indirect pathways were significant (β = 0.235 via locus of control; β = 0.159 via self-efficacy) and together exceed the direct effect. This configuration indicates that coaching shapes strategic posture chiefly by restructuring agentic cognition rather than by transmitting behaviour directly. While previous literature has explored the isolated effects of coaching or psychological traits on entrepreneurial outcomes, limited research has integrated these constructs into a holistic explanatory framework, especially in university-based incubation in emerging economies.

The findings that business coaching positively influences founders’ locus of control, self-efficacy, and entrepreneurial orientation are consistent with SCT, which posits that individuals learn and internalize behavioural patterns through observation, guided interaction, and socially supported feedback ( Bandura, 2001). Coaching provides the reflective environment that strengthens founders’ belief that outcomes stem from their own actions—a hallmark of internal locus of control—aligning with Tseng et al. (2022). The coaching relationship, characterized by trust, constructive feedback, and modelling of entrepreneurial reasoning, reinforces founders’ belief in their capabilities, mirroring ( Ndlovu-Hlatshwayo & Msimango-Galawe, 2023). The influence of coaching on entrepreneurial orientation aligns with prior findings that coaching supports strategic thinking, innovation, and proactive behaviour ( Mohamed, 2024; Daradkeh & Mansoor, 2023).

The measurement model also speaks to *which* facets of coaching matter most. Among the four dimensions, satisfaction (outer weight 0.485) and results (0.395) contributed more strongly to the business-coaching construct than session focus (0.269) and role clarity (0.221). This pattern suggests that, for student founders, the perceived value and tangible outcomes of coaching shape entrepreneurial cognition more than procedural features such as role definition or session structure. It also cautions against treating coaching as a homogeneous input: its effect is likely contingent on qualitative factors not captured here—coach competence, mentor–founder compatibility, coaching intensity, psychological safety, and domain-specific expertise. We therefore interpret the coaching construct as an aggregate whose influence is driven disproportionately by outcome- and satisfaction-related experiences, and we flag the disentangling of these contingencies as a priority for future research.

The study also demonstrates that locus of control and self-efficacy significantly influence entrepreneurial orientation, validating these personal cognitive factors as central drivers of strategic entrepreneurial behaviour. Founders with an internal locus of control exhibit stronger entrepreneurial orientation because they perceive themselves as capable of influencing outcomes, engaging more actively in risk-taking, innovation, and proactive opportunity-seeking—consistent with Hamzah and Othman (2023) and Leonelli et al. (2025). Self-efficacy also emerged as a significant predictor, reinforcing its theoretical role as a mechanism shaping willingness to pursue challenging entrepreneurial tasks, echoing Newman et al. (2019) and Ye and Kang (2025).

The mediation results provide deeper insight into the psychological mechanisms through which coaching influences entrepreneurial orientation. The significant mediating effect of locus of control confirms that coaching strengthens founders’ internal attributions, thereby enhancing strategic entrepreneurial behaviour, supporting SCT’s assertion that environmental cues shape personal cognition, which in turn influences behavioural outcomes ( Bandura, 2001; Hamzah & Othman, 2023). The mediating role of self-efficacy underscores how coaching enhances capability beliefs that subsequently drive entrepreneurial orientation, consistent with Setyawati and Geraldo (2021) and Caliendo et al. (2023). The comparatively weak direct effect, set against the significant indirect pathways, reinforces a cognitive-transformation reading: coaching does not merely provide skills—it reshapes the deeper cognitive structures that determine how founders behave entrepreneurially. That the attributional belief (locus of control) carried a larger indirect effect than the capability belief (self-efficacy) suggests that, for student founders, the shift from external to internal attribution may be the more consequential cognitive change.

### 5.2 Theoretical implication

This study extends Social Cognitive Theory in the context of university-based startup incubation in three ways. First, by dissociating two agentic beliefs, it shows that the mediating “person” node is not a single confidence factor: an attributional belief (locus of control) carried more of coaching’s effect than a capability belief (self-efficacy), indicating that the two cognitive mechanisms are not substitutable and should be modelled separately. Second, the dominance of the indirect over the direct pathway specifies a *cognitive-transformation* account of coaching rather than a *behavioural-transmission* account—a refinement that isolated-effect studies cannot make, and one that shows structured environmental inputs shape entrepreneurial behaviour chiefly by altering cognitive beliefs ( Bandura, 2001; Ndlovu-Hlatshwayo & Msimango-Galawe, 2023; Tseng et al., 2022). Third, the emerging-economy incubation context conditions the mechanism: where institutional support, industry linkages, and role models are comparatively thin, coaching may be one of few reliable sources of mastery experience and verbal persuasion, which could explain why its effect on cognition is pronounced. In more mature ecosystems, alternative environmental inputs (dense networks, experienced peers) may substitute for coaching, so the relative weight of the coaching pathway is itself context-dependent ( Donbesuur et al., 2020; Rosado-Cubero et al., 2024). Collectively, the study demonstrates that business coaching functions as a psychologically embedded mechanism that shapes entrepreneurial cognition and, ultimately, strategic entrepreneurial behaviour.

### 5.3 Practical implication

For universities, the results highlight the importance of embedding structured business coaching into broader entrepreneurial ecosystem initiatives. Since coaching enhances locus of control, self-efficacy, and entrepreneurial orientation, universities should institutionalize coaching-based interventions as a core component of campus entrepreneurship programs. For university business incubator managers, the evidence emphasizes designing incubation models that prioritize developmental coaching rather than merely technical or infrastructural support; the finding that satisfaction- and results-related facets weigh most heavily suggests that investment in outcome-focused, high-quality coaching—mentor matching, structured feedback cycles, and experiential workshops—will most effectively strengthen entrepreneurial cognition. For coaches, the study underscores their role in shaping entrepreneurial cognition through mastery experiences, vicarious learning, and motivational reinforcement, and the value of professional development that combines technical and psychological coaching skills. For young university entrepreneurs, active engagement in coaching can accelerate strategic orientation, risk-taking capacity, and innovation readiness; students should treat coaching not merely as advisory sessions but as platforms for cognitive transformation.

## 6. Conclusion, limitation, and future research

This study examined how business coaching within university business incubators influences the cognitive and strategic capacities of student founders in Indonesia through the mechanisms of locus of control, self-efficacy, and entrepreneurial orientation. Business coaching exerts a positive and significant effect on locus of control, self-efficacy, and entrepreneurial orientation; locus of control and self-efficacy each positively affect entrepreneurial orientation; and both cognitive beliefs significantly mediate the relationship between coaching and entrepreneurial orientation, with the indirect pathways collectively exceeding the direct effect.

The findings are specific to student-founded ventures in Indonesian university business incubators and were obtained from cross-sectional, self-reported data. They should not be read as evidence of universal coaching effects. Within this developing-economy, university-incubation setting, business coaching operated primarily by strengthening founders’ internal locus of control and self-efficacy, which in turn predicted entrepreneurial orientation, while the direct behavioural effect of coaching was comparatively modest. Because entrepreneurial ecosystems, institutional capabilities, and incubation models differ across national contexts—and because cultural differences in perceptions of control, confidence, and risk-taking may alter the psychological processes examined—generalization to non-university incubators, private accelerators, or developed-economy ecosystems should be made with caution, and any causal reading of these associations awaits further evidence.

Several avenues for future research emerge. First, comparative cross-country studies—especially between developing and developed ecosystems—would reveal contextual contingencies in how coaching influences entrepreneurial cognition and orientation. Second, future research could integrate additional psychological or environmental constructs, such as resilience, opportunity recognition, or institutional support quality. Third, longitudinal designs would allow scholars to examine how coaching-induced cognitive changes evolve and translate into long-term outcomes such as venture survival, growth, or innovation performance. Finally, expanding the sampling frame to include non-university incubators or private accelerators would clarify how coaching functions across diverse incubation models.

## Data Availability

Zenodo. Extended Data: Research Questionnaire for The Impact of Business Coaching on Founder Confidence and Entrepreneurial Orientation in University Incubated Startups. https://doi.org/10.5281/zenodo.18059883 ( Kuswinardi et al., 2025). This project contains the following extended data:
• Research Questionnaire – the complete questionnaire instrument used in this study, consisting of structured measurement items designed to capture respondents’ perceptions, attitudes, and behavioural responses in accordance with the study’s theoretical framework. The questionnaire serves as the primary data collection instrument and supports the empirical rigor of the research. • Underlying Anonymized Dataset – respondent-level demographic and survey data used for all descriptive and inferential analyses reported in the article. The dataset is fully anonymized and de-identified to comply with the study’s ethical approval requirements and to protect participant confidentiality. No personally identifiable information is included. All data and extended materials are available under the terms of the Creative Commons Attribution 4.0 International (CC BY 4.0) license.
