# Supplementary material for: The Impact of Business Coaching on Founder Confidence and Entrepreneurial Orientation in University Incubated Startups
Source: F1000Res. 2026 Sep 12;15:691. Originally published 2026 May 9. [Version 2] doi: 10.12688/f1000research.175927.2 (PMC13237547; doi:10.12688/f1000research.175927.2)
Supplement: Peer review history

# Reviewer report: Reviewer response for version 2

**DOI:** 10.5256/f1000research.205869.r520563

**Published:** 2026-09-17

**Authors:** Dian Arief Pradana

**Affiliations:** 1 Universitas 17 Agustus 1945 Banyuwangi, Banyuwangi, Indonesia

Thank you to the authors for the revisions and for their careful responses to the comments raised in the previous review. I have reviewed the revised manuscript and find that the authors have adequately addressed the comments and suggestions provided. The revisions have improved the clarity and overall quality of the manuscript.

Is the work clearly and accurately presented and does it cite the current literature?

Yes

If applicable, is the statistical analysis and its interpretation appropriate?

Yes

Are all the source data underlying the results available to ensure full reproducibility?

Yes

Is the study design appropriate and is the work technically sound?

Yes

Are the conclusions drawn adequately supported by the results?

Yes

Are sufficient details of methods and analysis provided to allow replication by others?

Partly

Reviewer Expertise:

My research interests include Educational Technology, Learning Technology, Curriculum and Teaching Materials Development, Entrepreneurship Education, Learning Management Systems (LMS), Flipped Classrooms, Project-Based Learning, digital learning innovation, and higher education research. My publications also focus on business incubation, entrepreneurial self-efficacy, adaptive learning systems, gamification in education, and technology integration in teaching and learning processes.

I confirm that I have read this submission and believe that I have an appropriate level of expertise to confirm that it is of an acceptable scientific standard.

# Reviewer report: Reviewer response for version 2

**DOI:** 10.5256/f1000research.205869.r520564

**Published:** 2026-09-16

**Authors:** Raya Sulistyowati, Muhammad Hasan

**Affiliations:** 1 Universitas Negeri Surabaya, Surabaya, Indonesia; 2 Universitas Negeri Makassar, Makassar, South Sulawesi, Indonesia

The authors have substantially addressed the main conceptual, methodological, and interpretive concerns raised in the previous review. The revised manuscript is clearer in defining “founder confidence” as an organizing term, provides greater detail on the sampling strategy, strengthens the connection with Social Cognitive Theory, and presents a more cautious interpretation of the findings. The limitations concerning cross-sectional data and generalizability are also appropriately acknowledged.

Is the work clearly and accurately presented and does it cite the current literature?

Partly

If applicable, is the statistical analysis and its interpretation appropriate?

Partly

Are all the source data underlying the results available to ensure full reproducibility?

Yes

Is the study design appropriate and is the work technically sound?

Partly

Are the conclusions drawn adequately supported by the results?

Yes

Are sufficient details of methods and analysis provided to allow replication by others?

Yes

Reviewer Expertise:

Research interests focus on strategic management, marketing, entrepreneurship, business education, organizational behavior, and digital transformation within modern organizations and technology-based entrepreneurial ecosystems. The research mainly explores digital marketing, consumer behavior, brand strategy, entrepreneurial orientation, business coaching, startup incubation, entrepreneurial self-efficacy, innovation capability, digital entrepreneurship, dynamic capabilities, and sustainable entrepreneurship. In addition, the studies examine the impact of artificial intelligence, organizational innovation, and digital transformation on employee productivity, marketing performance, business performance, change readiness, and sustainable competitive advantage. Research approaches commonly employ quantitative methods, Structural Equation Modeling (SEM/PLS-SEM), mediation analysis, and conceptual model development in the context of emerging economies.

We confirm that we have read this submission and believe that we have an appropriate level of expertise to confirm that it is of an acceptable scientific standard.

# Reviewer report: Reviewer response for version 1

**DOI:** 10.5256/f1000research.193950.r483901

**Published:** 2026-06-04

**Authors:** Dian Arief Pradana

**Affiliations:** 1 Universitas 17 Agustus 1945 Banyuwangi, Banyuwangi, Indonesia

Thank you for the opportunity to review this manuscript.

 This article examines the influence of business coaching on locus of control, self-efficacy, and entrepreneurial orientation in student startups participating in university business incubators in Indonesia using the PLS-SEM approach. The research topic is quite relevant, particularly in the context of developing countries that still need to strengthen university-based entrepreneurship incubation ecosystems. The integration of Social Cognitive Theory into the research model also has the potential to contribute to academic success. However, this article still has several recommendations for conceptual, methodological, and analytical improvements, requiring revision before it is suitable for indexing.

 a. Authors should provide a clear statement of the research gap at the beginning of the section, supported by a critical evaluation of previous works rather than a descriptive list.

 b. The literature review is informative but overly descriptive. It lists many studies without synthesizing how they relate to the manuscript reported in this article.

 c. The article utilizes current literature and systematically organizes the topics, but some sections are overly descriptive and repetitive. Claims of research novelty are also not sufficiently explained by providing theoretical gaps from previous research and model weaknesses in previous research that could ultimately be used to propose similar research in the university entrepreneurship incubation ecosystem.

 d. Although you have stated the source of the construct measurement, I recommend that you provide an explanation justifying the operationalization of the construct to measure a variable, and why the construct is appropriate for your research context. This can clarify the nature of the relationship between the indicators in the questionnaire to measure the variable and maximize construct validity.

 e. Because the study used purposive sampling, the potential for sampling bias is a significant issue. I recommend providing a description of the data regarding the number of universities/incubators involved, the incubator selection process (criteria), the distribution of respondents for each incubator, and an explanation of whether respondents are predominantly from a single university incubator or whether they are diverse. This will help clarify that this is a sample of startup founders in Indonesia, not just startup founders from a specific incubator.

 f. The presentation of the results and discussion should connect the data processing results to the theoretical framework.

Is the work clearly and accurately presented and does it cite the current literature?

Yes

If applicable, is the statistical analysis and its interpretation appropriate?

Yes

Are all the source data underlying the results available to ensure full reproducibility?

Yes

Is the study design appropriate and is the work technically sound?

Yes

Are the conclusions drawn adequately supported by the results?

Yes

Are sufficient details of methods and analysis provided to allow replication by others?

Partly

Reviewer Expertise:

My research interests include Educational Technology, Learning Technology, Curriculum and Teaching Materials Development, Entrepreneurship Education, Learning Management Systems (LMS), Flipped Classrooms, Project-Based Learning, digital learning innovation, and higher education research. My publications also focus on business incubation, entrepreneurial self-efficacy, adaptive learning systems, gamification in education, and technology integration in teaching and learning processes.

I confirm that I have read this submission and believe that I have an appropriate level of expertise to confirm that it is of an acceptable scientific standard, however I have significant reservations, as outlined above.

# Response

**Published:** 2026-07-08

**Authors:** Jacobus Wiwin Kuswinardi

**Affiliations:** School of Interdisciplinary Ma, Institut Teknologi Sepuluh Nopember, Surabaya, East Java, Indonesia

**I confirm that I have read this submission and believe that I have an appropriate level of expertise to confirm that it is of an acceptable scientific standard, however I have significant reservations, as outlined above.**

# Reviewer report: Reviewer response for version 1

**DOI:** 10.5256/f1000research.193950.r483899

**Published:** 2026-05-22

**Authors:** Deddy Marciano

**Affiliations:** 1 Universitas Surabaya, Surabaya, Indonesia

Overall, the paper demonstrates solid foundational quality and offers meaningful contextual insights into entrepreneurship development within emerging economies. The manuscript is well-structured, and the theoretical alignment between environmental support mechanisms, cognitive processes, and entrepreneurial behaviour is conceptually appropriate. The findings indicating that business coaching positively influences locus of control, self-efficacy, and entrepreneurial orientation, including the mediating effects of the two psychological constructs, provide valuable empirical contributions to the entrepreneurship and incubation literature.

 Nevertheless, several aspects of the manuscript would benefit from further refinement in order to strengthen its theoretical positioning, methodological rigor, and overall scholarly contribution expected at the level of an internationally competitive Q1 journal.

 One of the primary areas that requires further development concerns the articulation of the research gap and novelty. At present, the manuscript primarily argues that studies examining student startups, university incubators, and developing-country contexts remain limited. While this statement is valid, the gap is still framed rather broadly and descriptively. The manuscript would become substantially stronger if it more explicitly identified the unresolved theoretical issue being addressed. For instance, the authors may clarify whether the novelty lies in the psychological mechanisms through which coaching operates, the unique behavioural dynamics of student founders, the emerging-economy incubation context, or the dual mediating roles of locus of control and self-efficacy. In its current form, the contribution appears to emphasize “understudied context” rather than a more clearly defined theoretical advancement.

 In addition, the conceptualization of “founder confidence” would benefit from greater conceptual precision. The title highlights founder confidence as a central construct; however, the empirical model operationalizes this notion through locus of control and self-efficacy. These constructs, although related, represent conceptually distinct psychological dimensions. Self-efficacy reflects beliefs regarding one’s capability to perform entrepreneurial tasks, whereas locus of control concerns attributional beliefs regarding the source of outcomes. Accordingly, the manuscript should clarify whether founder confidence is intended as an umbrella psychological concept, a higher-order construct, or simply a general framing device used in the title. Greater conceptual clarity in this regard would enhance theoretical coherence.

 Another important consideration relates to the treatment of business coaching as a relatively homogeneous construct. The current model implicitly assumes that coaching exerts uniformly positive effects across participants. However, coaching effectiveness is often contingent upon several factors, including coach competence, mentor-founder compatibility, coaching intensity, psychological safety, and industry-specific expertise. Without acknowledging these qualitative dimensions, the construct risks becoming overly broad. Interestingly, the higher-order construct analysis suggests that satisfaction and perceived results contribute more strongly than role and session focus dimensions. This observation could be leveraged to develop a more nuanced discussion regarding which aspects of coaching are most influential in shaping entrepreneurial cognition and behaviour.

 Overall, this manuscript demonstrates promising potential and addresses an important area within entrepreneurship and incubation research. The contextual focus on Indonesian university incubators represents a valuable contribution, particularly given the limited empirical evidence from emerging economies. With further refinement of the theoretical framing, deeper conceptual articulation, additional methodological robustness, and more critical analytical discussion, the manuscript could make a stronger and more impactful contribution to the literature on entrepreneurial development and incubation ecosystems.

Is the work clearly and accurately presented and does it cite the current literature?

Yes

If applicable, is the statistical analysis and its interpretation appropriate?

Yes

Are all the source data underlying the results available to ensure full reproducibility?

Yes

Is the study design appropriate and is the work technically sound?

Yes

Are the conclusions drawn adequately supported by the results?

Yes

Are sufficient details of methods and analysis provided to allow replication by others?

Yes

Reviewer Expertise:

Finance and Innovation

I confirm that I have read this submission and believe that I have an appropriate level of expertise to confirm that it is of an acceptable scientific standard.

# Reviewer report: Reviewer response for version 1

**DOI:** 10.5256/f1000research.193950.r483904

**Published:** 2026-05-18

**Authors:** Raya Sulistyowati, Muhammad Hasan

**Affiliations:** 1 Universitas Negeri Surabaya, Surabaya, Indonesia; 2 Universitas Negeri Makassar, Makassar, South Sulawesi, Indonesia

General Assessment

 This manuscript investigates the impact of business coaching on locus of control, self-efficacy, and entrepreneurial orientation among student founders participating in university business incubators in Indonesia. Using Social Cognitive Theory (SCT) as the primary theoretical framework, the study examines both direct and indirect relationships among the variables through a mediation model tested using PLS-SEM analysis. The topic is relevant and timely, particularly given the increasing global attention toward university entrepreneurship ecosystems and incubation-based entrepreneurial development. The manuscript also demonstrates several strengths, including the use of recent literature, the application of a structured analytical approach, and the provision of open-access data through a public repository. Overall, the study contributes to the growing body of entrepreneurship research in emerging economies. Nevertheless, several important conceptual, methodological, and analytical aspects require further refinement before the article can be considered scientifically robust for publication.

 Clarity of Presentation and Literature Positioning

 The manuscript is generally well organized and follows a logical academic structure. The authors successfully integrate literature related to entrepreneurial orientation, business coaching, self-efficacy, and locus of control within the broader framework of Social Cognitive Theory. In addition, the manuscript incorporates many recent references published between 2022 and 2025, indicating that the authors are actively engaging with contemporary discussions in entrepreneurship and incubation research. However, despite these strengths, the manuscript still demonstrates conceptual inconsistencies that reduce clarity. Specifically, the terms founder confidence, self-efficacy, and entrepreneurial cognition are frequently used interchangeably throughout the manuscript without adequate conceptual distinction. This creates ambiguity regarding the actual focal construct of the study, especially because the title emphasizes founder confidence while the empirical model operationalizes self-efficacy and locus of control. The authors should therefore clearly define whether founder confidence represents a broader psychological construct, a synonym for self-efficacy, or a separate dimension altogether. Consistency across the title, theoretical framework, hypotheses, results, and discussion sections is essential to strengthen conceptual coherence.

 In addition, the novelty of the study should be articulated more explicitly. While the manuscript claims to address gaps related to entrepreneurial coaching and psychological mechanisms in university incubators, the literature review remains largely descriptive rather than critically synthetic. The authors summarize many prior studies but do not sufficiently explain what specific theoretical, methodological, or contextual gaps remain unresolved in previous research. Consequently, the unique contribution of this study is not yet fully convincing. To strengthen the manuscript, the authors should clearly identify how this research advances existing entrepreneurship incubation literature, particularly within emerging economy contexts such as Indonesia. More emphasis should be placed on explaining why the Indonesian incubation ecosystem provides a meaningful context for extending Social Cognitive Theory in entrepreneurial research.

 Study Design and Methodological Soundness

 The overall study design is appropriate for the stated research objectives. The use of a quantitative explanatory approach combined with Partial Least Squares Structural Equation Modeling (PLS-SEM) is suitable for examining the proposed mediation relationships among business coaching, locus of control, self-efficacy, and entrepreneurial orientation. The sample size of 220 respondents is acceptable and aligns with commonly recommended thresholds for PLS-SEM analysis. Furthermore, the manuscript demonstrates methodological rigor by evaluating reliability, convergent validity, discriminant validity, and collinearity through Cronbach’s alpha, composite reliability, AVE, HTMT, and VIF assessments. These procedures strengthen confidence in the technical adequacy of the measurement model.

 Despite these strengths, several methodological details require clarification. The manuscript provides limited information regarding the sampling procedure and participant recruitment process. Although purposive sampling is mentioned, the authors do not clearly explain how universities and business incubators were selected, how many institutions participated, or whether respondents originated from public or private universities across different Indonesian regions. Such information is important because entrepreneurial ecosystems may vary substantially depending on institutional and regional contexts. The manuscript would therefore benefit from a more transparent explanation of the sampling frame and participant recruitment strategy.

 Additionally, while the authors attempt to address common method bias using VIF values, the interpretation should be approached more cautiously. One VIF value reported in Table 5 is very close to the recommended threshold of 3.33, suggesting that the possibility of common method variance cannot be fully dismissed. The manuscript would be strengthened by incorporating additional assessments such as Harman’s single-factor test or marker variable analysis. Furthermore, the study does not discuss whether non-response bias was evaluated, which is particularly important in survey-based entrepreneurship research. Conducting and reporting an early-versus-late response comparison would improve the methodological robustness of the study.

 Measurement Transparency and Replicability

 The manuscript provides a relatively clear overview of the analytical procedures and measurement assessments employed in the study. The inclusion of publicly accessible datasets and questionnaires through Zenodo is a significant strength and demonstrates strong alignment with F1000Research open science principles. This level of transparency substantially enhances the reproducibility and credibility of the study.

 However, the manuscript still lacks sufficient detail regarding the development and adaptation of the research instruments. Although the authors mention that the questionnaire items were adapted from prior studies, the procedures used for adaptation are not adequately described. For instance, the manuscript does not explain whether the instruments underwent translation and back-translation processes, whether contextual modifications were made for Indonesian respondents, or whether pilot testing and expert validation were conducted before full-scale data collection. Such details are essential to establish the content validity and contextual appropriateness of the measurement instruments.

 Moreover, the SmartPLS estimation procedures are insufficiently documented. The manuscript does not specify important analytical parameters such as the number of bootstrap subsamples, the use of bias-corrected confidence intervals, or the resampling configuration applied during mediation testing. Providing these technical details would significantly improve the replicability of the study and allow other researchers to reproduce the analysis more accurately.

 Statistical Analysis and Interpretation

 The statistical analysis conducted in the manuscript is generally appropriate and competently executed. The use of PLS-SEM to test direct and indirect relationships is methodologically suitable given the mediation framework and higher-order constructs included in the study. The reliability and validity assessments are comprehensive, and the interpretation of the results is generally aligned with Social Cognitive Theory. The findings indicating that business coaching positively influences locus of control, self-efficacy, and entrepreneurial orientation are theoretically reasonable and supported by prior entrepreneurship literature.

 Nevertheless, some analytical aspects require deeper interpretation. One of the direct effects, namely the relationship between business coaching and entrepreneurial orientation, demonstrates a relatively weak T-statistic despite remaining statistically significant. This finding should be interpreted more cautiously because the relationship appears marginal compared to the stronger mediation pathways through locus of control and self-efficacy. The discussion section should therefore elaborate on why indirect psychological mechanisms may exert stronger influence than direct coaching effects. Such interpretation would enhance the theoretical sophistication of the manuscript.

 In addition, the manuscript focuses predominantly on statistical significance while giving limited attention to practical significance and effect magnitude. The inclusion of additional indicators such as effect size (f²), predictive relevance extensions, or PLSpredict assessments would provide a more comprehensive understanding of the model’s explanatory power. Expanding the discussion beyond p-values would also improve the managerial and theoretical implications of the study.

 Discussion and Theoretical Contribution

 The discussion section effectively connects the empirical findings with Social Cognitive Theory and entrepreneurship literature. The manuscript contributes to existing research by demonstrating that business coaching affects entrepreneurial orientation not only directly but also indirectly through psychological mechanisms such as locus of control and self-efficacy. This mediation perspective offers meaningful insight into how coaching interventions shape entrepreneurial cognition within university incubation environments.

 However, the theoretical contribution could be strengthened further. Much of the discussion focuses on confirming previous studies rather than critically extending theory or identifying nuanced insights emerging from the Indonesian context. The authors should elaborate more explicitly on how the findings refine or extend Social Cognitive Theory within entrepreneurship incubation settings. For example, the manuscript could discuss whether coaching primarily influences entrepreneurial behavior through cognitive transformation rather than direct behavioral intervention. Similarly, greater emphasis should be placed on explaining how entrepreneurial ecosystems in emerging economies differ from those in developed countries and how such differences may shape the effectiveness of business coaching. A deeper contextual discussion would significantly enhance the originality and theoretical contribution of the study.

 Conclusion and Practical Implications

 The conclusions presented in the manuscript are generally supported by the empirical findings. The study appropriately concludes that business coaching contributes positively to entrepreneurial orientation through the mediating roles of locus of control and self-efficacy. The practical implications directed toward universities, incubator managers, coaches, and student entrepreneurs are relevant and potentially valuable for improving entrepreneurship support systems within higher education institutions.

 Nonetheless, several conclusions appear somewhat generalized beyond the actual scope of the study. Since the research focuses exclusively on university business incubators in Indonesia, the authors should avoid making overly broad claims regarding universal applicability. Greater acknowledgment of contextual limitations would improve the scientific rigor of the conclusion section. In particular, the manuscript should emphasize that the findings are context-specific, cross-sectional in nature, and limited to university incubation settings within a developing economy.

 Data Availability and Research Transparency

 One of the strongest aspects of the manuscript is its commitment to research transparency and open science practices. The authors provide both the anonymized dataset and the research questionnaire through a publicly accessible Zenodo repository. This greatly enhances reproducibility, transparency, and scientific credibility. The data availability statement is clear and consistent with F1000Research indexing standards. Such openness should be acknowledged as a major strength of the study and reflects good research practice.

 Overall Recommendation

 Overall, this manuscript addresses an important and increasingly relevant issue in entrepreneurship and incubation research. The study demonstrates several strengths, including the use of recent literature, an appropriate analytical approach, and strong open-data practices. However, revisions are still required to improve conceptual consistency, methodological transparency, theoretical depth, and analytical interpretation. The most important revisions involve clarifying the conceptual meaning of founder confidence, strengthening the articulation of the study’s novelty, providing greater transparency regarding sampling and instrument adaptation procedures, and deepening the theoretical interpretation of the findings. Once these concerns are adequately addressed, the manuscript will make a valuable contribution to the entrepreneurship and university incubation literature.

 Recommendation: Minor Revision

Is the work clearly and accurately presented and does it cite the current literature?

Partly

If applicable, is the statistical analysis and its interpretation appropriate?

Partly

Are all the source data underlying the results available to ensure full reproducibility?

Yes

Is the study design appropriate and is the work technically sound?

Partly

Are the conclusions drawn adequately supported by the results?

Yes

Are sufficient details of methods and analysis provided to allow replication by others?

Yes

Reviewer Expertise:

Research interests focus on strategic management, marketing, entrepreneurship, business education, organizational behavior, and digital transformation within modern organizations and technology-based entrepreneurial ecosystems. The research mainly explores digital marketing, consumer behavior, brand strategy, entrepreneurial orientation, business coaching, startup incubation, entrepreneurial self-efficacy, innovation capability, digital entrepreneurship, dynamic capabilities, and sustainable entrepreneurship. In addition, the studies examine the impact of artificial intelligence, organizational innovation, and digital transformation on employee productivity, marketing performance, business performance, change readiness, and sustainable competitive advantage. Research approaches commonly employ quantitative methods, Structural Equation Modeling (SEM/PLS-SEM), mediation analysis, and conceptual model development in the context of emerging economies.

We confirm that we have read this submission and believe that we have an appropriate level of expertise to confirm that it is of an acceptable scientific standard, however we have significant reservations, as outlined above.

## References

[1] : Entrepreneurial self-efficacy: A systematic review of the literature on its theoretical foundations, measurement, antecedents, and outcomes, and an agenda for future research. *Journal of Vocational Behavior* .2019;110: 10.1016/j.jvb.2018.05.012403-419 10.1016/j.jvb.2018.05.012

[2] : Riding the Roller Coaster of Emotional Energy in a Student Startup Incubator. *Entrepreneurship Education and Pedagogy* .2025;8(4) : 10.1177/25151274241263077530-552 10.1177/25151274241263077

# Response

**Published:** 2026-07-08

**Authors:** Jacobus Wiwin Kuswinardi

**Affiliations:** School of Interdisciplinary Ma, Institut Teknologi Sepuluh Nopember, Surabaya, East Java, Indonesia

We sincerely thank the reviewers for their careful, consistent, and constructive evaluation, which has substantially strengthened the manuscript.

 In response, we have clarified the focal construct by defining *founder confidence* as an organizing term for two conceptually distinct agentic beliefs—internal locus of control (attributional) and entrepreneurial self-efficacy (capability)—each modelled as a separate mediator, and we ensured consistent terminology across the title, abstract, framework, hypotheses, results, and discussion.

 We sharpened the research gap and novelty to specify the theoretical problem addressed—whether coaching shapes strategic posture directly or through cognitive restructuring within the SCT triad—rather than a merely contextual gap. We expanded the sampling and instrument sections to report the number, type, and regional distribution of participating incubators and the instrument-adaptation procedure; strengthened the methodological rigor by reporting the full-collinearity assessment, Harman's single-factor test, a non-response-bias check, and the complete bootstrap configuration; and added effect sizes (f²) and a PLSpredict assessment alongside a more cautious interpretation of the modest direct coaching effect.

 Finally, we deepened the theoretical contribution by articulating how the findings extend Social Cognitive Theory and by calibrating the conclusions to the study's cross-sectional, single-country, university-incubation scope. We have addressed each specific comment point by point in the accompanying response, and we hope the reviewers find the revised version considerably improved.
